# Trichodermin exhibits potent anti-glioblastoma activity by inducing cell cycle arrest and apoptosis, suppressing invasion, and enhancing temozolomide efficacy

**DOI:** 10.1080/14756366.2026.2694181

**Published:** 2026-07-20

**Authors:** Hung-Pei Tsai, Tzong-Huei Lee, Hong-Liang Lin, Tzu-wei Lin, Wen-Hsin Chang, Chien-Ju Lin

**Affiliations:** ^a^ Division of Neurosurgery, Department of Surgery, Kaohsiung Medical University Hospital, Kaohsiung Medical University, Kaohsiung, Taiwan; ^b^ Regenerative Medicine and Cell Therapy Research Center, Kaohsiung Medical University, Kaohsiung, Taiwan; ^c^ Institute of Fisheries Science, National Taiwan University, Taipei, Taiwan; ^d^ School of Pharmacy, College of Pharmacy, National Defense Medical University, Taipei, Taiwan; ^e^ School of Pharmacy, College of Pharmacy, Kaohsiung Medical University, Kaohsiung, Taiwan; ^f^ Department of Sports Medicine, College of Medicine, Kaohsiung Medical University, Kaohsiung, Taiwan

**Keywords:** Trichodermin, glioblastoma, temozolomide, apoptosis, epithelial-mesenchymal transition

## Abstract

Trichodermin, a sesquiterpene antibiotic from *Trichoderma* species, shows anticancer potential. In this study, anti-glioblastoma activity was evaluated by (3-(4,5-di methyl thiazol-2-yl)-2,5-diphenyltetrazolium bromide) (MTT) assay, colony formation, lactate dehydrogenase (LDH) release assay, flow cytometry, wound-healing, transwell invasion, adhesion, Western blot, combination-index analysis, and an orthotopic luciferase glioblastoma mouse model. Trichodermin reduced viability and clonogenicity and increased LDH release of T98G and A172 cells. Trichodermin induced G_2_/M arrest with p53 activation and downregulation of cyclin B, cyclin A, and cyclin-dependent kinase 1 (CDK1). In addition, trichodermin induced caspase-dependent apoptosis. Invasion, wound healing, and adhesion were suppressed with modulation of epithelial-mesenchymal transition (EMT)-related proteins. Combination index analysis demonstrated a synergistic interaction between trichodermin and temozolomide, possibly due to increased apoptosis. In the mouse model, intraperitoneal trichodermin inhibited intracranial tumour growth and prolonged survival, and increased cleaved caspase-3 expression in tumour tissues. These findings indicate that trichodermin exerts anti-glioblastoma activity and warrants further preclinical evaluation as a potential adjunct to temozolomide therapy.

## Graphical Abstract

**Figure:**
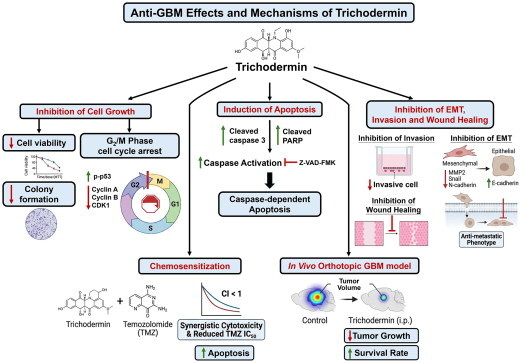

## 1. Introduction

Glioblastoma multiforme (GBM) is the most common and aggressive primary malignant brain tumour in adults, classified as a grade IV glioma according to the World Health Organisation (WHO).^1^ Originating from astrocytes, GBM is characterised by its highly invasive nature, rapid proliferation, and diffuse infiltration into adjacent brain tissue, which complicate surgical resection and contribute to poor prognosis.^2^ Despite advancements in medical interventions, the median survival time for patients with GBM remains approximately 15 months, with a 5-year survival rate of only 6.8%.^3^ This poor prognosis positions GBM among the most fatal systemic tumours, with a mortality rate second only to pancreatic and lung cancers.^4^ The current standard of care for GBM involves maximal safe surgical resection followed by adjuvant radiotherapy and chemotherapy with temozolomide (TMZ).^5^^,^^6^ TMZ, a second-generation alkylating agent, exerts its cytotoxic effects through DNA methylation, resulting in tumour cell apoptosis.^2^^,^^7^ However, its efficacy is significantly hampered by intrinsic and acquired resistance mechanisms, primarily driven by the DNA repair enzyme O6-methylguanine-DNA methyltransferase (MGMT).^8^ MGMT directly counteracts the therapeutic effects of TMZ by removing its cytotoxic methyl adducts, thereby restoring DNA integrity and promoting tumour cell survival.^8^ Consequently, approximately 55% of patients with GBM exhibit inherent resistance to TMZ, severely limiting the long-term effectiveness of this chemotherapeutic approach.^8^ Given the aggressive nature of GBM and the high incidence of treatment resistance, disease recurrence risk is very high, with up to 90% of patients experiencing tumour relapse within the first year following initial treatment.^9^ Moreover, recurrent tumours often exhibit a more aggressive phenotype, further diminishing treatment efficacy and survival outcomes.^9^ Despite extensive research efforts, no improvement in GBM prognosis has been achieved in the past two decades, underscoring the urgent need for novel therapeutic strategies.^9^

Trichodermin is a naturally occurring sesquiterpene antibiotic derived from *Trichoderma spp.* and other fungal species. It has a potent antifungal activity and can inhibit protein synthesis in eukaryotic cells by targeting the ribosomal peptidyl transferase centre.^10^ The compound was first identified in the 1960s and has since been extensively studied for its antimicrobial, antifungal, and anticancer properties.^11^^,^^12^ Trichodermin also demonstrates some anticancer potential. For example, in human chondrosarcoma cells, trichodermin triggers endoplasmic reticulum (ER) stress-mediated apoptosis by upregulating key stress proteins such as inositol-requiring enzyme 1 (IRE1), phosphorylated protein kinase RNA-like ER kinase (p-PERK), and glucose-regulated protein 78 (GRP78), leading to cytochrome *c* release and caspase activation.^11^ Trichodermin induces apoptosis via c-Jun N-terminal kinase activation and DNA damage in pancreatic cancer cells, particularly those harbouring *p53* mutations.^13^ Similarly, trichodermin has been shown to cause G_0_/G_1_ cell cycle arrest and tumour growth suppression of ovarian cancer.^14^ In addition, trichodermin inhibits oral cancer cell migration and invasion by targeting matrix metalloproteinases and histone deacetylase-2 (HDAC-2)-mediated signalling pathways.^15^ These findings suggest that trichodermin and its derivatives hold promise as therapeutic agents in oncology, warranting further investigation into their clinical applications.

Despite its promising anticancer properties, the effects of trichodermin on GBM have not been investigated. Given GBM’s highly invasive nature, resistance to standard therapies, and poor prognosis, there remains an urgent need for novel therapeutic strategies. This study aims to evaluate the efficacy of trichodermin in GBM and to elucidate its underlying mechanisms of action. By investigating its effects on GBM cell proliferation, apoptosis, metastasis, and related signalling pathways, we aim to determine the potential of trichodermin as a therapeutic agent for this aggressive brain tumour.

## 2. Materials and methods

### 2.1. Chemicals and reagents

Trichodermin was isolated and purified from the solid-state fermented products of *Trichoderma brevicompactum* NTU439 and kindly provided by one of the co-authors (THL). The physical characteristics and NMR spectra of trichodermin were consistent with those reported previously.^16^ The purity of the pure isolate was higher than 95%, as confirmed by the 1H and 13 C NMR spectra. Dulbecco’s modified eagle medium (DMEM), minimum essential medium (MEM), foetal bovine serum (FBS), and polyvinylidene fluoride (PVDF) membrane were obtained from Cytiva (Marlborough, MA). Pen-Strep-AmphoB solution was purchased from Sartorius (Gottingen, Germany). Ribonuclease A (RNase A), TMZ, Annexin V-FITC/propidium iodide (PI) apoptosis detection kit, and Triton X-100 were purchased from Sigma-Aldrich (St. Louis, MO). N-benzyloxycarbonyl-val-ala-asp(O-methyl) fluoromethyl ketone (Z-VAD-FMK) was purchased from Calbiochem (San Diego, CA). Lactate dehydrogenase (LDH) release assay kit (CytoTox 96^®^ Non-Radioactive Cytotoxicity Assay) was purchased from Promega (Madison, WI). Transwell inserts (SPLInsert^™^ Hanging) were purchased from SPL Life Sciences (Gyeonggi-do, Korea). Antibodies against p-p53, p-53, cyclin B, cyclin A, CDK1, caspase 3, cleaved caspase 3, PARP, Snail, and anti-mouse and anti-rabbit IgG horseradish peroxidase (HRP)-conjugated secondary antibodies were purchased from Cell Signaling (Danvers, MA). Anti-N-cadherin and E-cadherin antibodies were purchased from Proteintech (Rosemont, IL). Anti-matrix metalloproteinase-2 (MMP2) antibody was purchased from Abcam (Cambridge, UK). The RIPA buffer, enhanced chemiluminescence (ECL), anti-β-actin antibody, and 3-[4,5-dimethylthiazol-2-yl]-2,5-diphenyltetrazolium bromide (MTT) were purchased from Millipore (Billerica, MA).

### 2.2. Cell culture

Human GBM cell lines, T98G (CRL-1690) and A172 (CRL-1620), were obtained from the American Type Culture Collection (ATCC, Manassas, VG). Cells were cultured in minimum essential medium (MEM) (for T98G cells) and Dulbecco’s modified Eagle medium (DMEM) (for A172 cells), supplemented with 10% foetal bovine serum (FBS), 1% penicillin/streptomycin/amphotericin B, and 1% L-glutamine. Cells were maintained in a humidified incubator at 37 °C with 5% CO_2_ and were routinely tested for mycoplasma contamination. Experiments were conducted using logarithmically growing cells.

### 2.3. Cell viability assay

The effect of trichodermin on GBM cell viability was assessed using the MTT assay. Briefly, T98G and A172 cells were seeded in 96-well plates at a density of 5 × 10³ cells per well and allowed to adhere overnight. Cells were then treated with increasing concentrations of trichodermin (0–40 μM) for 24, 48, or 72 h. Following treatment, 0.5 mg/mL MTT solution was added to each well and incubated at 37 °C for 4 h. The resulting formazan crystals were dissolved in dimethyl sulfoxide, and absorbance was measured at 570 nm using a microplate reader (Bio-Tek Instruments Inc., Winooski, VT). Data were normalised to the control group, and cell viability was expressed as a percentage.

### 2.4. Cytotoxicity assay

Lactate dehydrogenase (LDH) release was quantified to assess trichodermin-induced cytotoxicity. T98G and A172 cells (5 × 10³ cells per well) were seeded in 96-well plates and treated with various concentrations of trichodermin (0–40 μM) for 72 h. After treatment, culture supernatants were collected, and LDH activity was measured using an LDH cytotoxicity assay kit following the manufacturer’s instructions. Briefly, 50 μL of supernatant from each well was transferred to a new 96-well plate and mixed with 50 μL of CytoTox 96^®^ Reagent. The mixtures were incubated for 30 min at room temperature in the dark. The reaction was then terminated by adding 50 μL of Stop solution. Absorbance was measured at 490 nm using a microplate reader (Bio-Tek Instruments Inc., Winooski, VT), and cytotoxicity was calculated using the following formula

$$\begin{array}{l} \text{Cytotoxicity}\left(\text{\%}\right)=\\ \left(\text{Experimental LDH release }-\text{ Spontaneous LDH release}\right)/\left(\text{Maximum LDH release }-\text{ Spontaneous LDH release}\right)\times 100 \end{array}$$

where maximum LDH release was determined by lysing cells with Triton X-100.

### 2.5. Colony formation assay

To evaluate the long-term effects of trichodermin on GBM cell proliferation, a colony formation assay was performed. Cells were seeded in 6-well plates at a density of 1000 cells per well and treated with different concentrations of trichodermin (0–5 μM). After 10–14 days, colonies were fixed with 4% paraformaldehyde and stained using 0.5% crystal violet. Colonies containing at least 50 cells were counted, and the percentage of colony formation was calculated relative to the control group.

### 2.6. Cell cycle analysis

Cell cycle distribution was analysed using propidium iodide (PI) staining and flow cytometry. T98G and A172 cells were seeded in 6-well plates at a density of 1 × 10^5^ cells per well and were treated with drugs. Trichodermin-treated cells were harvested and fixed in 70% ethanol at 4 °C overnight. After washing with phosphate buffered saline (PBS), cells were incubated with RNase A (100 μg/mL) and PI (40 μg/mL) in the dark for 30 min at 37 °C. DNA content was analysed using a flow cytometer, and cell cycle distribution was determined using CXP software V2.3 (Beckman Coulter, Brea, CA).

### 2.7. Apoptosis analysis

Apoptosis was detected using an Annexin V-FITC/PI apoptosis detection kit following the manufacturer’s instructions. Cells (1 × 10^5^ cells per well) seeded in 6-well plates were treated with trichodermin (0–40 μM) for 72 h or 5 μM trichodermin for 24, 48, and 72 h, collected, and resuspended in a binding buffer. Annexin V-FITC and PI were added and incubated for 15 min at room temperature in the dark. Stained cells were analysed using a flow cytometer, and the percentage of apoptotic cells was quantified using CXP software (Beckman Coulter, Brea, CA).

### 2.8. Wound healing assay

The wound healing assay was used to evaluate the effect of trichodermin on collective wound closure. Cells were seeded in 12-well plates at a density of 3 × 10^5^ cells per well and allowed to reach 90% confluency. A linear wound was gently created using a sterile pipette tip, and detached cells were removed by washing with PBS. Cells were then incubated in serum-free medium containing trichodermin (0, 1, or 5 μM). Images were captured at 0, 6, 12, 24, and 30 h using an inverted microscope, and wound closure was quantified using MShot Image Analysis System 1.5.2 software (Guangzhou Micro-shot Technology Co., Guangzhou, China).

### 2.9. Invasion assay

The invasive potential of GBM cells was evaluated using Matrigel-coated transwell inserts with an 8-μm pore size membrane (SPLInsert^™^ Hanging). Each insert consists of an upper and a lower chamber separated by a porous membrane coated with Matrigel. After treatment with trichodermin (0–40 μM) for 72 h, cells (1 × 10^4^ cells per insert) were suspended in serum-free medium and re-seeded into the upper chamber of the transwell inserts. The lower chamber contained a complete medium with 10% FBS as a chemoattractant. Cells were then allowed to invade through the Matrigel layer and porous membrane towards the lower chamber. After 24 h of incubation, non-invading cells on the upper surface of the membrane were gently removed. Cells that had invaded through the membrane and attached to the lower membrane surface were fixed, stained using crystal violet, and counted under a microscope.

### 2.10. Cell adhesion assay

Cell adhesion was evaluated by plating GBM cells onto Matrigel-coated 12-well plates. After treatment with trichodermin (0–40 μM) for 72 h, the treated cells were harvested and re-seeded in 12-well plates (1 × 10^4^ cells/well). Subsequently, the cells were washed with PBS to remove non-adherent cells after incubation for 1 and 24 h. Adherent cells were fixed, stained with crystal violet, and photographed using an inverted microscope. The number of adherent cells were calculated, and the percentage was calculated relative to the control group.

### 2.11. Western blot analysis

Protein expression levels were assessed using Western blot. Cells were seeded in 6-cm dishes at a density of 3 × 10^5^ cells per dish and were treated with drugs. At the end of treatment, cells were lysed in RIPA buffer containing protease and phosphatase inhibitors, and protein concentration was determined using a bicinchoninic acid assay. Equal amounts (50 μg/lane) of protein were separated using 10% or 12% (particularly for pro-caspase 3 and cleaved-caspase 3) SDS-PAGE, transferred to PVDF membranes, and blocked with 5% skim milk for 1 h. Membranes were incubated overnight at 4 °C with primary antibodies against p-p53 (1:500), p53 (1:1000), cyclin B (1:1000), cyclin A (1:1000), CDK1 (1:1000), caspase-3 (1:2000), cleaved caspase 3 (1:1000), PARP (1:1000), MMP2 (1:1000), Snail (1:1000), N-cadherin (1:2000), E-cadherin (1:1000), and β-actin (1:20000) followed by incubation with corresponding HRP-conjugated anti-mouse or anti-rabbit secondary antibodies (1:1000) at room temperature for 1 h. Protein bands were visualised using enhanced chemiluminescence (ECL) reagent and photographed using a MultiGel-21 Image System (TOP BIO CO., New Taipei City, Taiwan). The density of the bands was quantified using ImageJ software.

### 2.12. Combination index (CI) analysis

Cells were treated with TMZ at 100, 200, 400, 800, and 1000 μM alone or in combination with trichodermin (1, 2.5, and 5 μM) for 72 h, and cell viability was assessed using the MTT assay. The results from MTT assay were applied for combination index (CI) analysis. CompuSyn software was used to determine the combinational effect between trichodermin and TMZ, the CI values were interpreted as follows: CI < 1 indicates synergism, CI = 1 indicates an additive effect, and CI > 1 indicates antagonism. The IC_50_ values of TMZ, either as a monotherapy or in combination with trichodermin, were determined via nonlinear regression analysis of the MTT assay data.

### 2.13. Animal model

To evaluate the *in vivo* efficacy of trichodermin, six-week-old male athymic nude mice purchased from BioLASCO (BioLASCO Taiwan Co., Ltd., Taiwan) (body weight 25 g) were used to establish an orthotopic glioblastoma xenograft model. This immunodeficient strain was chosen to permit stable engraftment of human GBM cells and to enable longitudinal, non-invasive monitoring of intracranial tumour burden by bioluminescence imaging. Mice were acclimatised for at least 7 days and housed under specific pathogen-free conditions with a 12 h light/dark cycle, with *ad libitum* access to standard chow and sterilised water. Animals were group-housed (3 mice/cage) in ventilated cages with appropriate bedding and environmental enrichment. The number of animals was minimised and justified *a priori*, resulting in a total of *n* = 30 mice. Mice were randomly allocated into three groups: vehicle control (*n* = 10), trichodermin 0.1 mg/kg/day (*n* = 10), and trichodermin 0.5 mg/kg/day (*n* = 10). The dosing regimen used in this study was determined based on previously published studies evaluating the anticancer activity of trichodermin.^11^^,^^13^ For intracranial implantation, mice were anaesthetised with isoflurane (induction 2%, maintenance 1.5% in oxygen). Luciferase-expressing T98G cells (1 × 10^5^ cells in 5 μL) were stereotactically injected into the right striatum at coordinates 1 mm (AP), 1 mm (ML), and 3 mm (DV) relative to bregma. Body temperature was maintained using a heating pad during the procedure. Animals were monitored until full recovery and subsequently at least once per day for 7 days. Supportive care was provided as needed to minimise suffering. Beginning on day 7 post-implantation, mice received daily intraperitoneal injections of trichodermin. Tumour progression was assessed by bioluminescence imaging using an IVIS system (Xenogen) after intraperitoneal administration of D-luciferin under brief anaesthesia (2% isoflurane). Imaging was performed on days 7, 14, and 21. Bioluminescence intensity was quantified to assess intracranial tumour burden. Body weight was recorded on days 0, 7, 14, and 21 as a general indicator of treatment tolerability. For survival analysis, mice were followed until the predefined humane endpoint or the planned study endpoint. In accordance with animal welfare requirements, survival time was defined as the interval from tumour implantation to the day of humane euthanasia rather than the time of spontaneous death. Humane endpoints included >20% body-weight loss, persistent neurological deficits (such as circling and seizures), impaired ambulation, inability to eat/drink, or a moribund condition. Mice reaching endpoints or at the planned study endpoint were euthanized in accordance with institutional guidelines. Animals were euthanized using carbon dioxide (CO_2_) inhalation in a dedicated euthanasia chamber. CO_2_ was introduced gradually at a displacement rate of approximately 20% of the chamber volume per minute, in accordance with the guidelines of the Laboratory Animal Center of Kaohsiung Medical University. Death was confirmed by the cessation of respiration and heartbeat, followed by cervical dislocation as a secondary physical method to ensure complete euthanasia. All the procedures involving animals were conducted in compliance with the ARRIVE guidelines (Animal Research: Reporting of *In Vivo* Experiments) and were approved by the Institutional Animal Care and Use Committee.

### 2.14. Haematoxylin and eosin staining and immunohistochemical staining

At the experimental endpoint, mice were euthanized according to the approved animal protocol, and brain tissues were collected. The excised brain tissues were fixed by immersion in 4% paraformaldehyde, processed for paraffin embedding, and sectioned at a thickness of 3 μm. For haematoxylin and eosin (H&E) staining, paraffin-embedded brain tumour sections were deparaffinized in xylene and rehydrated through a graded ethanol series. The sections were stained with haematoxylin, rinsed with running tap water, and counterstained with eosin. After dehydration through graded ethanol solutions and clearing in xylene, the sections were mounted with a permanent mounting medium. Histological morphology of intracranial tumour tissues was examined under a light microscope, and images were captured at 100× and 400× magnification. For immunohistochemical analysis, paraffin-embedded sections were deparaffinized in xylene and rehydrated through graded ethanol solutions. Antigen retrieval was performed using citrate buffer (pH 6.0). Endogenous peroxidase activity was blocked with 3% hydrogen peroxide, followed by blocking of non-specific binding. The sections were then incubated with an anti-cleaved caspase-3 primary antibody (Cell Signalling Technology; #9664) (1:500) for 1 h at room temperature. After washing, the sections were incubated with a polymer-based HRP detection system (Dako, Glostrup, Denmark; K4003) for 30 min at room temperature. Immunoreactivity was visualised using 3,3′-diaminobenzidine (Dako, Glostrup, Denmark; K5007) as the chromogen, followed by counterstaining with haematoxylin. Finally, the sections were dehydrated, cleared, mounted, and examined under a light microscope.

### 2.15. Statistical analysis

At least three independent experiments were conducted for each assay. Data were presented as mean ± standard deviation (SD). Statistical comparisons between groups were performed using one-way analysis of variance followed by Tukey’s *post hoc* test. Differences were considered statistically significant when the *p* values were less than 0.05. All statistical analyses were performed using GraphPad Prism software. The exact *p* values are provided in the supplementary material (Table S1).

## 3. Results

### 3.1. Trichodermin exhibits cytotoxic effects and inhibits colony formation in GBM cells T98G and A172

**Figure 1.**
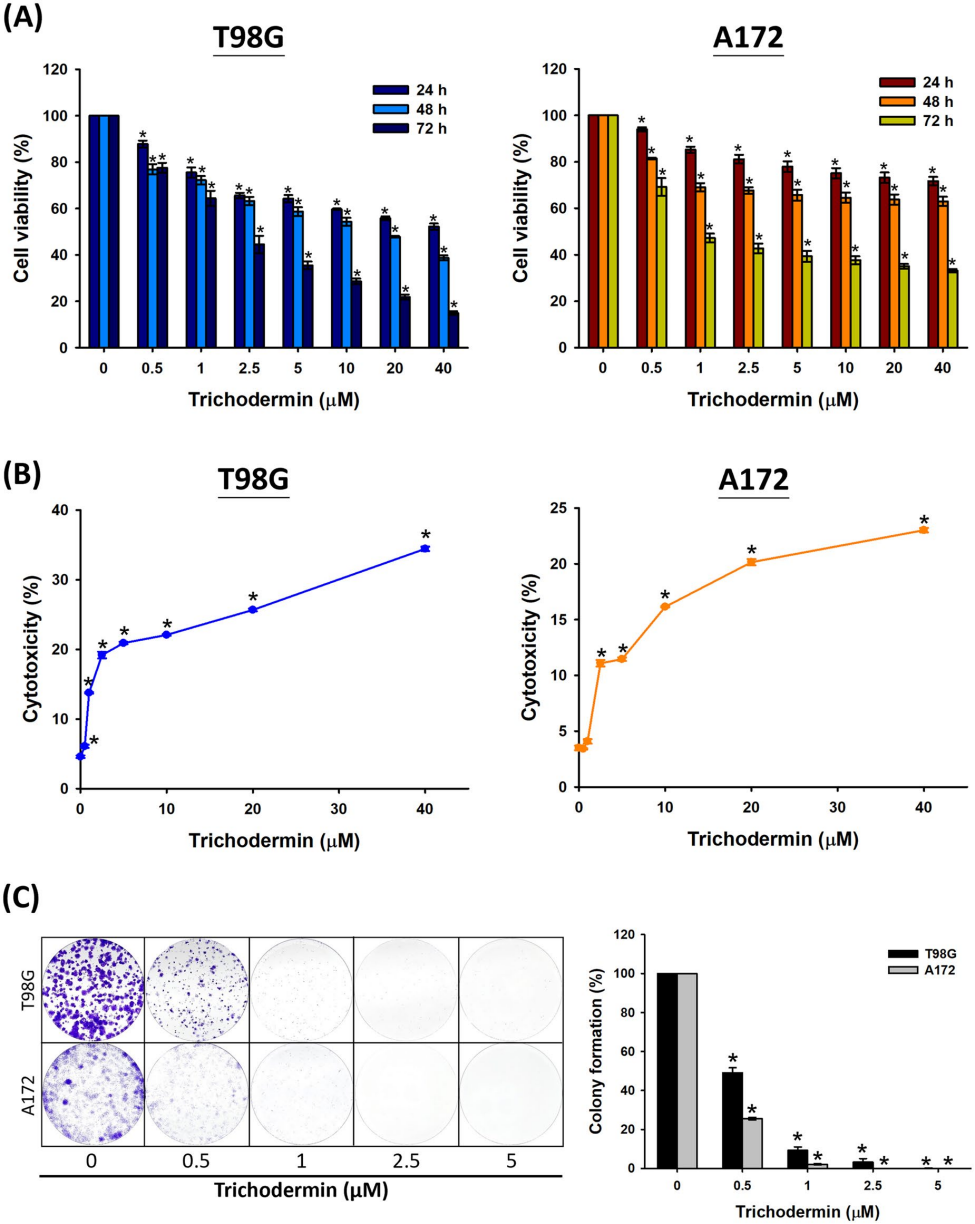
Trichodermin inhibits glioblastoma cell growth and increases cytotoxicity. (A) T98G and A172 glioblastoma cells were treated with trichodermin (0–40 µM) for 24, 48, and 72 h. Cell viability was assessed using the MTT assay. (B) Cytotoxicity was evaluated using the LDH release assay after 72 h of trichodermin treatment. (C) The colony formation ability of T98G and A172 cells was assessed by treating 1000 cells per well with low concentrations of trichodermin (0–5 µM) followed by incubation for 14 days. Colonies were stained, counted, and quantified. Data are presented as mean ± SD for *n* = 3. *p < 0.05 vs. control.

To evaluate the cytotoxic effects of trichodermin on GBM cells, T98G and A172 cells were treated with increasing concentrations of the compound, and their viability, cytotoxicity, and colony-forming ability were assessed. The results showed that trichodermin significantly reduced cell viability in a dose- and time-dependent manner (*p* < 0.05). In T98G cells, exposure to trichodermin led to a progressive decline in viability, with a more pronounced effect observed at higher concentrations, particularly at 10 μM and above (Figure 1A). A similar effect was observed in A172 cells, although they exhibited slightly lower sensitivity to the treatment compared to T98G. To further investigate the cytotoxic effects of trichodermin, a cytotoxicity assay was performed, revealing a dose-dependent increase in cell death (*p* < 0.05). In T98G cells, cytotoxicity reached approximately 34.4% at the highest concentration tested (40 μM), whereas A172 cells exhibited a lower response, with cytotoxicity levels peaking at around 23.0% (Figure 1B). These findings suggest that while both GBM cell lines are susceptible to trichodermin, T98G cells may be more sensitive to its cytotoxic effects. The ability of GBM cells to form colonies following trichodermin treatment was also evaluated. Colony formation was significantly inhibited in both cell lines in a dose-dependent manner (*p* < 0.05). Even at low concentrations, such as 0.5 μM, a substantial reduction in colony numbers was observed. At concentrations of 2.5 μM and higher, colony formation was completely inhibited, indicating that trichodermin effectively impairs the clonogenic ability of GBM cells (Figure 1C). Quantification of colony formation confirmed that both T98G and A172 cells exhibited a similar pattern of inhibition, with A172 cells displaying slightly greater resistance. Overall, these results demonstrate that trichodermin exerts potent anti-GBM effects by reducing cell viability, increasing cytotoxicity, and strongly inhibiting colony formation. These findings suggest that trichodermin may serve as a promising therapeutic candidate for GBM treatment.

### 3.2. Trichodermin induces G_2_/M phase arrest in GBM cells via activation of p53 and downregulation of cyclin B, cyclin A, and CDK1

**Figure 2.**
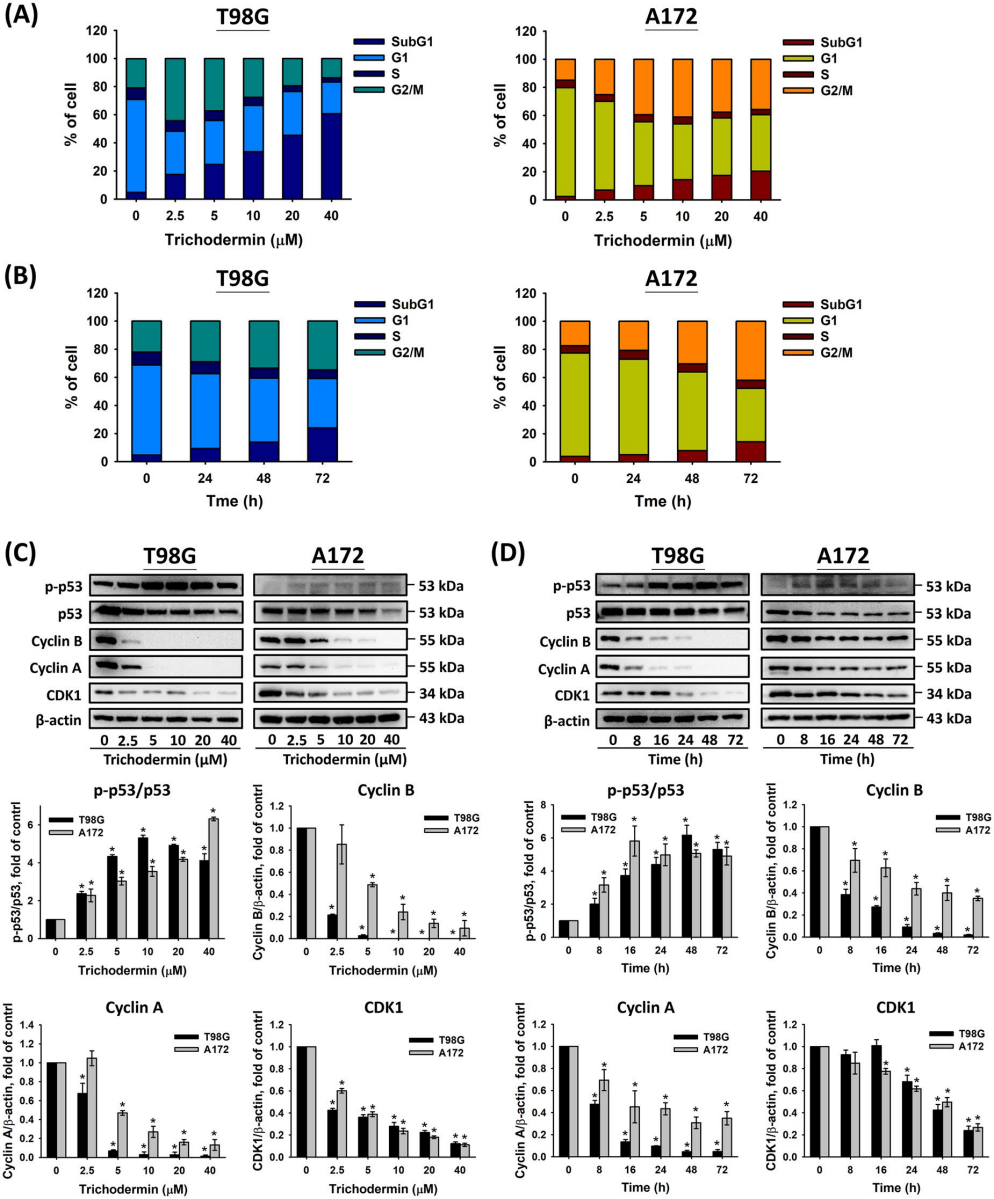
Trichodermin induces G_2_/M cell cycle arrest in glioblastoma cells. (A and B) T98G and A172 glioblastoma cells were treated with trichodermin (0–40 µM) for 72 h (A) or with 5 µM trichodermin for 0–72 h (B). Cell cycle distribution was analysed using PI staining followed by flow cytometry. (C and D) Western blot analysis was performed to examine the expression levels of p-p53, p53, cyclin B, cyclin A, and CDK1 in T98G and A172 cells treated with trichodermin (0–40 µM) for 72 h (C) or with 5 µM trichodermin for 0–72 h (D). Data are presented as mean ± SD for *n* = 3. *p < 0.05 vs. control.

To further investigate the effects of trichodermin on cell cycle regulation in GBM cells, flow cytometry analysis and Western blot were performed to examine cell cycle distribution and the expression of key regulatory proteins in T98G and A172 cells. Cell cycle analysis revealed that trichodermin treatment induced significant alterations in cell cycle distribution. In both T98G and A172 cells, increasing concentrations of trichodermin led to an accumulation of cells in the G_2_/M phase, accompanied by a reduction in the G_1_ and S phase populations (*p* < 0.05) (Figure 2A). This effect was particularly evident at higher doses (≥5 μM), suggesting that trichodermin induces G_2_/M phase arrest. A time-course analysis further confirmed this trend, showing a progressive increase in the G_2_/M phase population after 24–72 h of treatment (*p* < 0.05) (Figure 2B). To further elucidate the molecular mechanisms underlying trichodermin-induced G_2_/M arrest, the expression levels of key cell cycle regulators, including phosphorylated-p53 (p-p53), p53, cyclin B, cyclin A, and CDK1, were examined using Western blot. The dose-dependent increase of p-p53 and decreases in cyclin B, cyclin A, and CDK1 expression were observed in both T98G and A172 cells following trichodermin treatment (*p* < 0.05) (Figure 2C), indicating a strong inhibitory effect on G_2_/M phase progression. Similarly, a time-dependent change in protein expression was observed, with significant reductions observed as early as 8–16 h post-treatment, which persisted through 72 h (*p* < 0.05) (Figure 2D). These findings suggest that trichodermin effectively disrupts GBM cell cycle progression by inducing G_2_/M arrest, possibly through the activation of p53 and downregulation of cyclin B, cyclin A, and CDK1. The inhibition of these key regulators reinforces the potential of trichodermin as an anti-GBM agent by impairing cell cycle progression and preventing tumour cell proliferation.

### 3.3. Trichodermin induces caspase-dependent apoptosis in GBM cells through activation of caspase-3 and PARP cleavage

**Figure 3.**
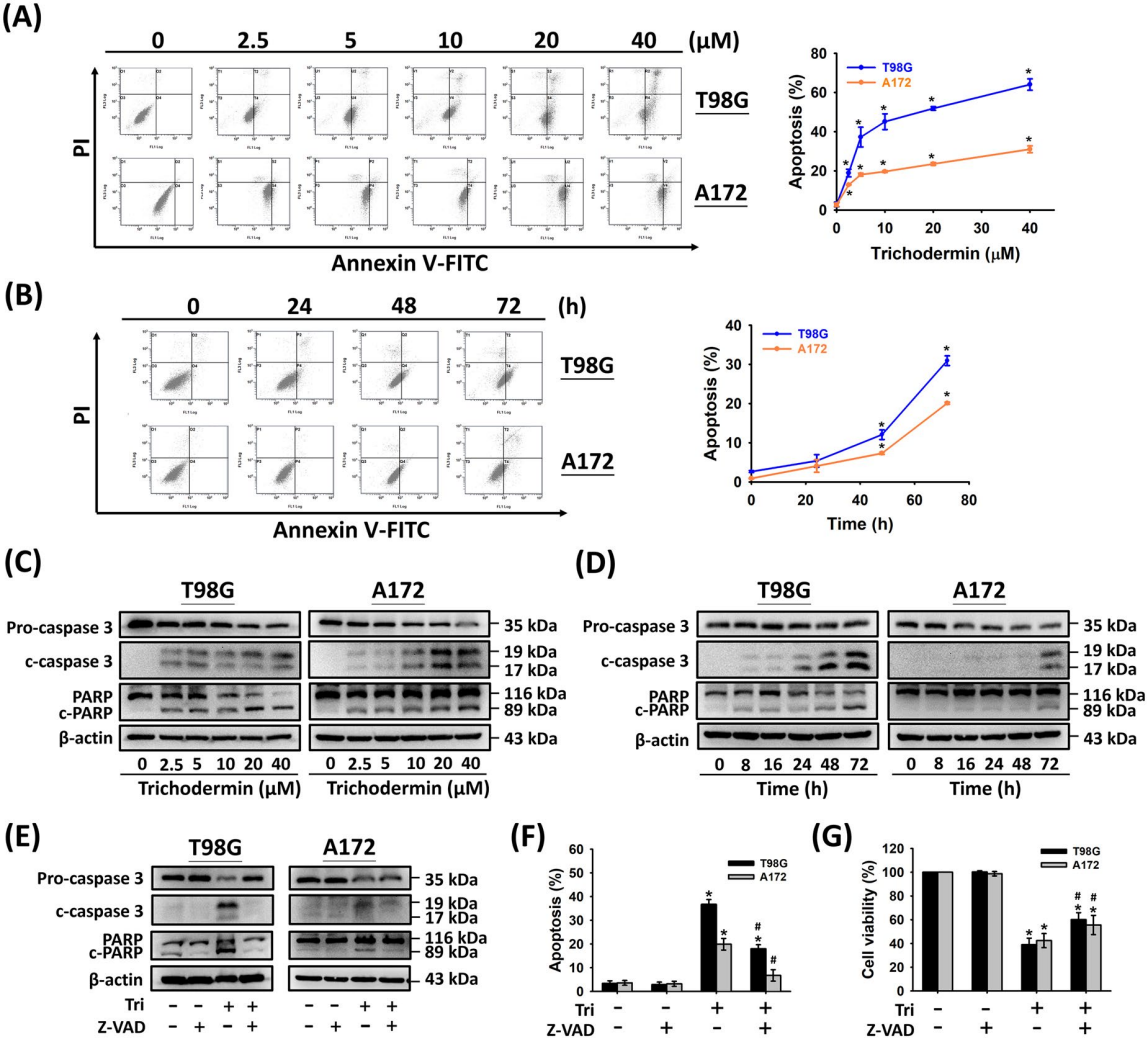
Trichodermin induces caspase-dependent apoptosis in glioblastoma Cells. (A and B) T98G and A172 glioblastoma cells were treated with trichodermin (0–40 µM) for 72 h (A) or with 5 µM trichodermin for 24, 48, and 72 h (B). Apoptosis was analysed using Annexin V-FITC and PI staining followed by flow cytometry. (C and D) Western blot analysis was performed to examine the expression levels of pro-caspase-3, cleaved caspase-3 (c-caspase-3), PARP, and cleaved PARP (c-PARP) in T98G and A172 cells treated with trichodermin (0–40 µM) for 72 h (C) or with 5 µM trichodermin for 0–72 h (D). (E–G) T98G and A172 cells were pre-treated with 100 µM z-VAD followed by 5 µM trichodermin (Tri) for 72 h. Western blot analysis was performed to detect pro-caspase-3, cleaved caspase-3 (c-caspase-3), PARP, and cleaved PARP (c-PARP) (E). Apoptosis was assessed using Annexin V-FITC and PI staining followed by flow cytometry (F), and cell viability was determined using the MTT assay (G). Data are presented as mean ± SD for *n* = 3. *p < 0.05 vs. control; ^#^*p* < 0.05 vs. trichodermin-treated group. Tri, trichodermin.

To determine whether trichodermin induces apoptosis in GBM cells, apoptosis levels were assessed using Annexin V/PI staining. The results revealed that trichodermin treatment significantly increased apoptosis in both T98G and A172 cells in a dose- and time-dependent manner. Annexin V/PI staining demonstrated a clear increase in apoptotic cell populations following trichodermin treatment. As the concentration increased from 2.5 to 40 μM, apoptosis levels progressively increased (*p* < 0.05), with T98G cells exhibiting a more pronounced response than A172 cells (Figure 3A). Similarly, a time-course analysis indicated that apoptosis increased with prolonged exposure to trichodermin, reaching the highest levels at 72 h in both cell lines (*p* < 0.05) (Figure 3B). To further investigate the molecular mechanisms underlying trichodermin-induced apoptosis, Western blot analysis was performed to assess the activation of key apoptotic markers. Trichodermin treatment resulted in a dose-dependent cleavage of caspase-3 and PARP in both T98G and A172 cells, indicating caspase-dependent apoptosis (Figure 3C). This effect was also observed in a time-dependent manner, where cleaved caspase-3 and cleaved PARP levels progressively increased over time (Figure 3D). The pan-caspase inhibitor Z-VAD-FMK was used to confirm the role of caspase activation in trichodermin-induced apoptosis. Pre-treatment with Z-VAD-FMK effectively blocked caspase-3 and PARP cleavage, demonstrating that trichodermin-induced apoptosis is caspase-dependent (Figure 3E). Moreover, Annexin V/PI staining showed that Z-VAD-FMK significantly reduced apoptosis in both T98G and A172 cells compared to trichodermin treatment alone (*p* < 0.05) (Figure 3F). A corresponding increase in cell viability was also observed in the presence of Z-VAD-FMK, further confirming that trichodermin-induced cell death is mediated through caspase-dependent apoptosis (*p* < 0.05) (Figure 3G). Overall, these findings indicate that trichodermin effectively induces apoptosis in GBM cells through a caspase-dependent mechanism, with T98G cells exhibiting greater sensitivity to treatment compared to A172 cells. These results suggest that trichodermin may serve as a potential therapeutic agent for GBM by triggering apoptotic pathways.

### 3.4. Trichodermin suppresses invasion and wound healing in GBM cells by modulating EMT-related proteins and reducing MMP2 and Snail expression

**Figure 4.**
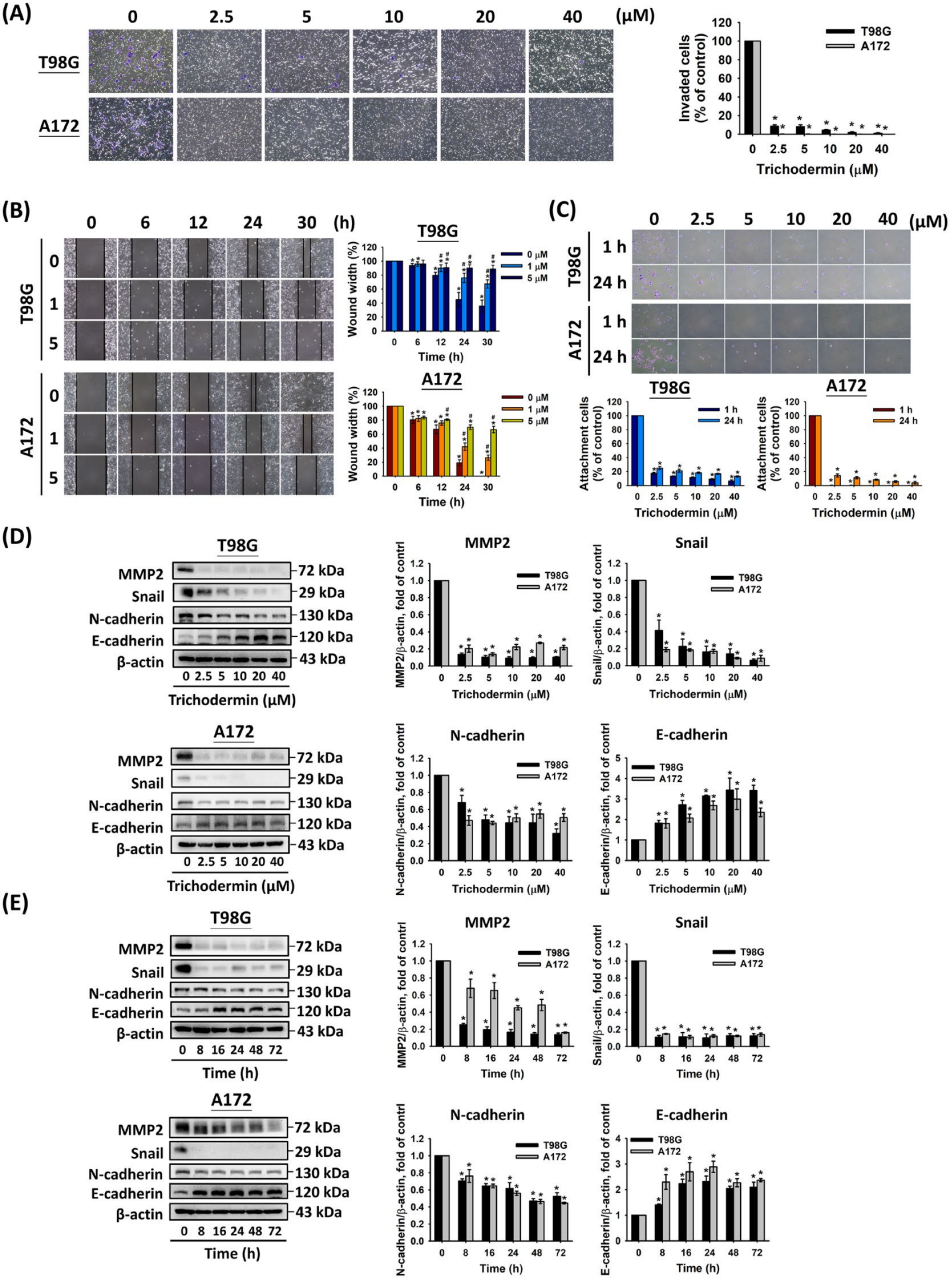
Trichodermin suppresses invasion, wound healing, and adhesion in glioblastoma Cells. (A) T98G and A172 glioblastoma cells were treated with trichodermin (0–40 µM) for 72 h, then reseeded (1 × 10^4^ cells/well) into transwell chambers for an invasion assay. After 24 h, cells that invaded through the membrane were stained with crystal violet, imaged, and quantified to determine the relative invasion percentage. (B) Wound healing assay was used to assess the effect of trichodermin on wound closure. T98G and A172 cells were treated with 0, 1, or 5 µM trichodermin for up to 30 h. Wound areas were monitored at different time points using phase-contrast microscopy, and wound closure width was measured. (C) Adhesion ability was evaluated by treating T98G and A172 cells with trichodermin (0–40 µM) for 72 h, followed by reseeding into 12-well plates (1 × 10^4^ cells/well). After 1 and 24 h, adherent cells were stained with crystal violet, imaged, and quantified. (D and E) Western blot analysis was performed to assess the expression levels of metastasis-related proteins, including MMP2, Snail, N-cadherin, and E-cadherin. T98G and A172 cells were treated with trichodermin (0–40 µM) for 72 h (D) or with 5 µM trichodermin for 0–72 h (E). Data are presented as mean ± SD for *n* = 3. *p < 0.05 vs. control; #*p* < 0.05 vs. trichodermin-treated group.

To evaluate the impact of trichodermin on the invasive and wound healing properties of GBM cells, a series of functional assays were conducted in T98G and A172 cells. The results demonstrated that trichodermin effectively suppresses GBM cell invasion and motility in the dose- and time-dependent manner. To assess the effects of trichodermin on GBM cell invasion, a Matrigel invasion assay was performed. Trichodermin treatment resulted in a substantial decrease in the number of invasive cells, with significant inhibition observed at concentrations from 2.5 μM and above (*p* < 0.05) (Figure 4A). The wound healing assay confirmed that trichodermin-treated cells exhibited a significant delay in wound closure time. Both cell lines showed a dose-dependent inhibition of cell motility, with reduced wound closure at 12 and 24 h post-treatment and near-complete inhibition at 30 h, especially in T98G cells (*p* < 0.05) (Figure 4B). In addition, the attachment ability of cancer cells was evaluated by the adhesion assay. T98G and A172 cells treated with 0–40 μM trichodermin for 72 h were re-seeded on 12-well plates (1 × 10^4^ cells/well) and cultured for 1 h and 24 h. The number of attached cells was significantly decreased by trichodermin at concentrations as low as 2.5 μM (*p* < 0.05) (Figure 4C). These findings suggest that trichodermin effectively impairs the motility, invasive, and adhesion capabilities of GBM cells. To explore the molecular mechanisms underlying trichodermin-induced inhibition of invasion and motility, Western blot analysis was conducted to examine the expression of key epithelial-mesenchymal transition (EMT)-related proteins. The results showed that trichodermin treatment led to a dose-dependent downregulation of MMP2 and Snail (*p* < 0.05), both of which are known to promote invasion and metastasis. Additionally, a decrease in N-cadherin and a concurrent increase in E-cadherin expression were observed (*p* < 0.05), indicating a shift towards a more epithelial-like, less invasive phenotype (Figure 4D). A time-course analysis further confirmed these effects, showing progressive reductions in MMP2, Snail, and N-cadherin expression, along with increased E-cadherin levels, over a 72-h period (*p* < 0.05) (Figure 4E). Taken together, these findings demonstrate that trichodermin effectively inhibits GBM cell invasion and suppresses wound healing ability by modulating EMT-related pathways. The suppression of MMP2, Snail, and N-cadherin, coupled with the upregulation of E-cadherin, suggests that trichodermin impairs the invasive potential of GBM cells, highlighting its potential as a therapeutic agent targeting GBM progression.

### 3.5. Trichodermin synergistically enhances TMZ-induced cytotoxicity in GBM cells

**Figure 5.**
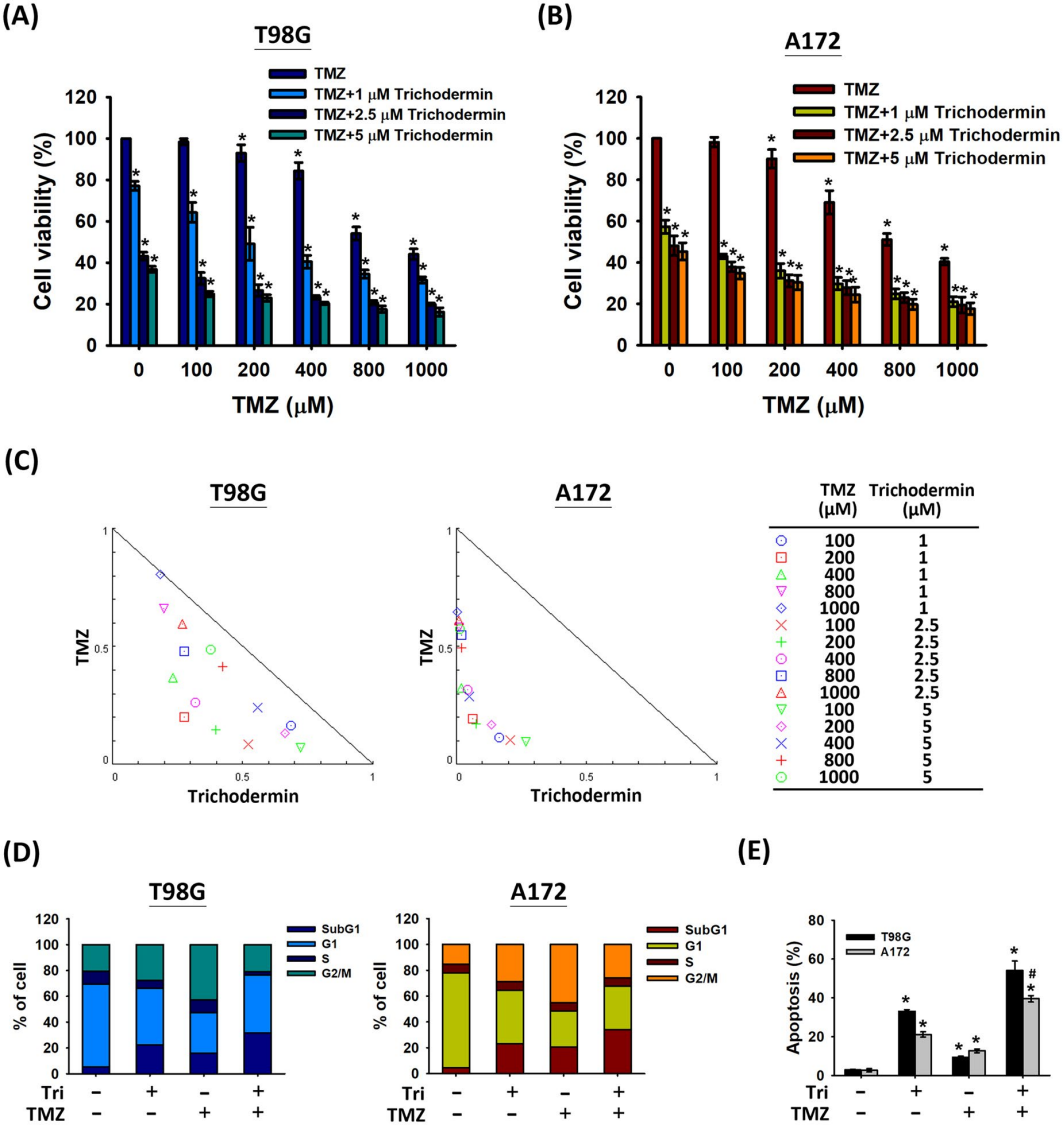
Trichodermin synergistically enhances the effects of temozolomide in glioblastoma Cells. (A and B) T98G (A) and A172 (B) glioblastoma cells were treated with low doses of trichodermin (1, 2.5, and 5 µM) in combination with increasing concentrations of temozolomide (TMZ) (0, 100, 200, 400, 800, and 1000 µM) for 72 h. Cell viability was measured using the MTT assay. (C) The combination index (CI) was calculated using CompuSyn software to evaluate the drug interaction effects. CI values indicate a synergistic effect when CI < 1. (D and E) T98G and A172 glioblastoma cells were treated with 5 µM trichodermin and 800 µM TMZ alone or in combination for 72 h. Cell cycle distribution (D) and apoptosis (E) were respectively analysed using PI staining or Annexin V-FITC/PI staining followed by flow cytometry. Data are presented as mean ± SD for *n* = 3. *p < 0.05 vs. control; ^#^*p* < 0.05 vs. TMZ-treated group. Tri, trichodermin; TMZ, temozolomide.

To evaluate the potential synergistic effects of trichodermin in combination with TMZ in GBM cells, cell viability was assessed in T98G and A172 cells treated with various concentrations of TMZ alone or in combination with trichodermin. The results demonstrated that co-treatment with trichodermin significantly enhanced the cytotoxic effects of TMZ in both cell lines. In T98G cells, TMZ alone reduced cell viability in a dose-dependent manner; however, when combined with increasing concentrations of trichodermin (1, 2.5, and 5 μM), a further decrease in viability was observed at all tested TMZ concentrations (*p* < 0.05) (Figure 5A). A similar trend was observed in A172 cells, where the combination treatment led to a more pronounced reduction in cell viability compared to TMZ alone (*p* < 0.05) (Figure 5B). Based on the data of viability assay, the IC_50_ concentrations of TMZ in both TMZ alone and combined with trichodermin were calculated. After combination with various concentrations of trichodermin, the IC_50_ value was further decreased in dose-dependent manner as compared with TMZ alone (Table 1). These findings indicate that trichodermin enhances the cytotoxicity of TMZ in GBM cells. To determine whether the interaction between TMZ and trichodermin was synergistic, the data of MTT assays were subjected to analyse the CI values using CompuSyn software. The CI plot showed that all data points fell below the additive effect line in both T98G and A172 cells, suggesting a synergistic interaction between trichodermin and TMZ (Figure 5C). The results confirmed that the combination treatment exhibited a synergistic effect across multiple concentrations, particularly at lower TMZ doses, where trichodermin significantly enhanced TMZ-induced cytotoxicity. To further investigate the cellular basis of this synergistic interaction, cell cycle distribution and apoptosis were analysed by flow cytometry after treatment with 5 μM trichodermin and/or 800 μM TMZ for 72 h. As shown in Figure 5(D), trichodermin or TMZ treatment alone induced G_2_/M phase accumulation, whereas the combination of trichodermin and TMZ reduced the proportion of cells in the G_2_/M phase. Notably, co-treatment markedly increased the subG_1_ population compared with either treatment alone, suggesting enhanced induction of cell death. Consistently, apoptosis analysis revealed that the combination treatment significantly increased the percentage of apoptotic cells compared with TMZ treatment alone (*p* < 0.05) (Figure 5E). Overall, these findings suggest that trichodermin enhances the cytotoxicity of TMZ in GBM cells through a synergistic interaction, which is associated with a shift from cell cycle arrest towards increased apoptotic cell death. This result highlights the potential therapeutic benefit of combining trichodermin with standard chemotherapy in glioblastoma treatment.

**Table 1.** Combination of trichodermin with temozolomide (TMZ) decreases the IC_50_ values of TMZ.

Cell line	IC_50_ (μM) of TMZ at different concentration of Trichodermin			
	0 μM	1 μM	2.5 μM	5 μM
T98G	873.6 ± 6.5	486.2 ± 17.1*	155.5 ± 9.8*	74.1 ± 11.0*
A172	813.5 ± 17.5	279.1 ± 9.1*	217.4 ± 3.0*	178.5 ± 18.9*

Each value represents the mean ± SD for *n* = 3.

*p < 0.05 vs. control.

### 3.6. Trichodermin suppresses GBM tumour growth and prolong survival rate in an orthotopic mouse model in a dose-dependent manner

**Figure 6.**
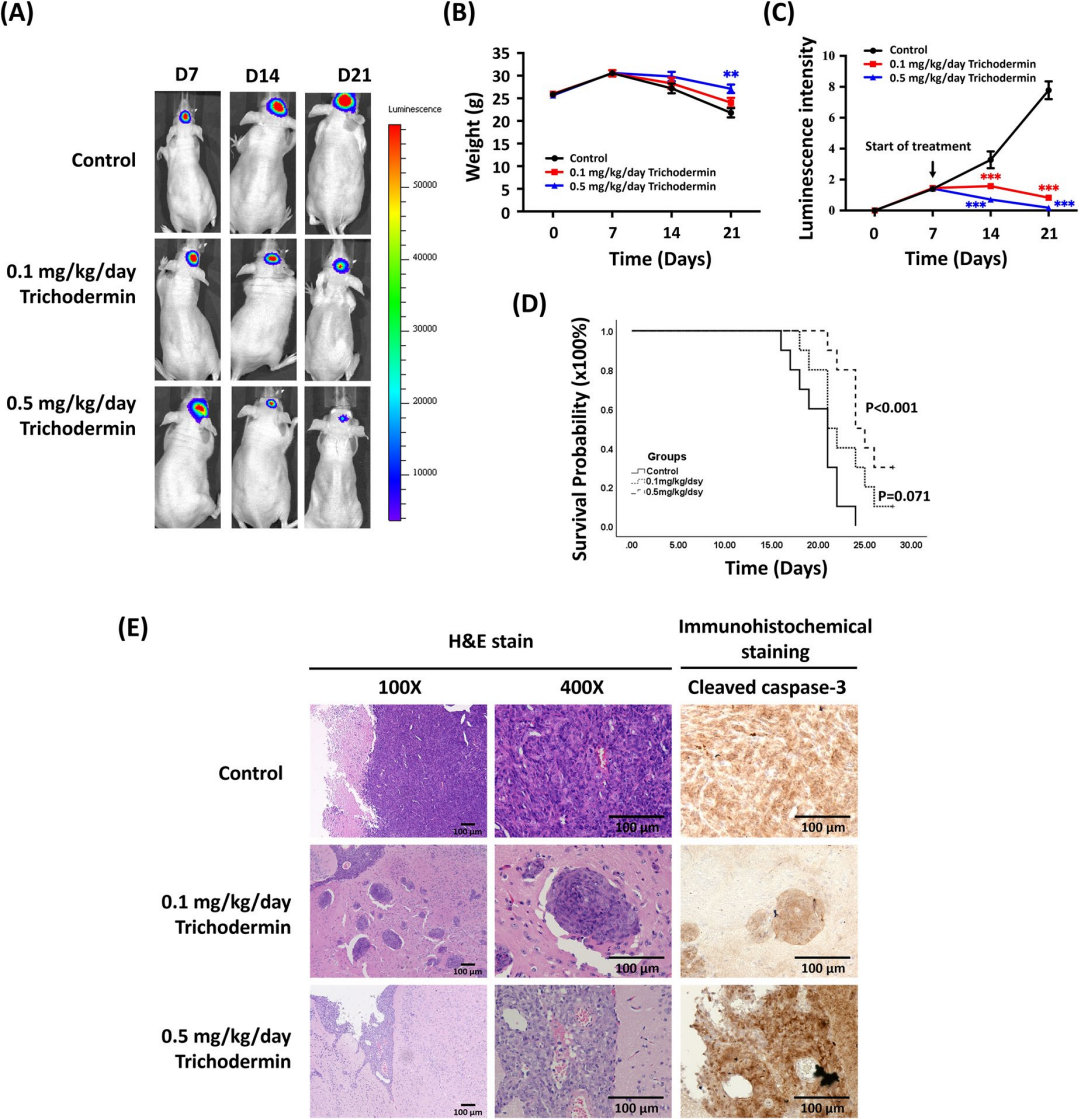
Trichodermin suppresses intracranial tumour growth and prolongs survival in an intracranial glioblastoma orthotopic model. (A) Six-week-old nude mice were intracranially injected with luciferase-expressing T98G cells to establish tumours. Starting on day 7 after implantation, mice received daily intraperitoneal injections of vehicle, 0.1 mg/kg/day trichodermin, or 0.5 mg/kg/day trichodermin. Representative IVIS bioluminescence images obtained on days 7, 14, and 21 are shown. (B) Changes in body weight during treatment. (C) Quantitative analysis of bioluminescence intensity showing suppression of intracranial tumour growth by trichodermin. The arrow indicates the start of treatment on day 7. (D) Kaplan–Meier curves based on time to humane endpoint/euthanasia in the vehicle control and trichodermin-treated groups. The 0.5 mg/kg/day trichodermin group showed a significant survival benefit compared with the control group (*p* < 0.001), whereas the 0.1 mg/kg/day group showed a non-significant effect (*p* = 0.071). Data are presented as mean ± SD for *n* = 10. **p < 0.01 and ***p < 0.001 vs. the respective control. (E) Representative H&E staining (100× and 400× magnification) and immunohistochemical staining for cleaved caspase-3 in intracranial tumour tissues from vehicle control, 0.1 mg/kg/day trichodermin, and 0.5 mg/kg/day trichodermin groups. Scale bars, 100 μm.

To evaluate the *in vivo* efficacy of trichodermin in GBM, an orthotopic mouse model was established using luciferase-expressing GBM cells. Mice were treated with either 0.1 or 0.5 mg/kg/day of trichodermin, starting from day 7 post-inoculation. Tumour progression was monitored using bioluminescence imaging at days 7, 14, and 21. Representative bioluminescence images showed that intracranial tumour signals increased progressively in the control group from days 7 to 21, whereas both trichodermin-treated groups exhibited weaker signals, with the greatest suppression observed in the 0.5 mg/kg/day group (Figure 6A). Body-weight monitoring revealed a gradual decline in the control group over the course of the experiment, whereas body weight was better maintained in trichodermin-treated mice; notably, the 0.5 mg/kg/day group showed significantly higher body weight than the control group on day 21 (*p* = 0.003; Figure 6B). Quantitative analysis of bioluminescence intensity further demonstrated that tumour burdens were comparable among groups on day 7 before treatment initiation, but were significantly lower in both trichodermin-treated groups on days 14 and 21 than in the control group (both *p* < 0.001; Figure 6C), indicating effective suppression of intracranial tumour progression. The Kaplan-Meier survival analysis showed that the 0.1 mg/kg/day group exhibited a trend towards prolonged survival that did not reach statistical significance (*p* = 0.071; Figure 6D), whereas the 0.5 mg/kg/day group showed a significant survival benefit compared with the control group (*p* < 0.001; Figure 6D). Histological examination was performed to evaluate the effects of trichodermin *in vivo*. Haematoxylin and eosin (H&E) staining showed densely packed tumour cells in the control group, whereas tumours from trichodermin-treated mice exhibited reduced tumour cellularity and increased tissue disruption, with these changes being more pronounced in the 0.5 mg/kg/day group. In addition, immunohistochemical staining for cleaved caspase-3 demonstrated apoptosis-associated staining in trichodermin-treated tumour tissues, particularly in the high-dose group (Figure 6E). Collectively, these findings indicate that trichodermin suppresses orthotopic GBM growth *in vivo*, promotes apoptosis-related tumour cell death, and that the higher dose confers a clear survival advantage in this model.

## 4. Discussion

This study investigated the multifaceted anti-tumour properties of trichodermin against GBM, demonstrating its ability to inhibit tumour proliferation, induce apoptosis, suppress invasion and wound closure ability, and enhance the efficacy of TMZ. Trichodermin effectively reduced GBM cell viability in dose- and time-dependent manners. T98G and A172 cells exhibited a marked decrease in survival following exposure to increasing concentrations of trichodermin, with T98G cells displaying greater sensitivity. The LDH release assay further confirmed trichodermin-induced cytotoxicity, particularly at higher concentrations, while the suppression of colony formation suggested that trichodermin significantly impaired the clonogenic potential of GBM cells. These observations align with the results of previous studies in which trichodermin exhibited cytotoxic effects in other malignancies, including chondrosarcoma and pancreatic cancer.^11^^,^^13^ Cell cycle analysis revealed that trichodermin treatment induced significant alterations in cell cycle progression, leading to an increased proportion of cells arrested in the G_2_/M phase. Western blot analysis confirmed that trichodermin downregulated key regulators of mitotic progression, including cyclin B, cyclin A, and CDK1, and upregulated the phosphorylation of p53 in dose- and time-dependent manners. These results implied that the compound disrupts cell cycle control by preventing mitotic entry. Similar G_2_/M arrest has been observed in previous studies involving trichodermin and other natural compounds such as curcumin and resveratrol, both of which exhibit anti-proliferative effects in GBM cells through cell cycle modulation.^17^^,^^18^ Trichodermin significantly increased apoptosis percentage in GBM cells, as demonstrated by annexin V/PI staining and the cleavage of caspase-3 and PARP. Further validation using the caspase inhibitor Z-VAD-FMK revealed that trichodermin-induced apoptosis was caspase-dependent. These findings are consistent with previous reports of trichodermin-induced apoptosis in other malignancies, including ovarian and oral cancers, where the compound triggered caspase activation and mitochondrial dysfunction.^14^^,^^15^ Additionally, the ability of trichodermin to promote apoptosis through ER stress and mitochondrial dysfunction has been well-documented in chondrosarcoma models.^11^

The highly invasive nature of GBM presents a major obstacle in treatment, as infiltrating tumour cells contribute to recurrence and resistance to therapy.^19–21^ Herein, trichodermin significantly inhibited GBM cell invasion and suppressed wound healing, as evidenced by transwell invasion and wound healing assays. Western blot analysis revealed that trichodermin downregulated MMP2 and Snail, two key regulators of invasion, while simultaneously reducing N-cadherin and increasing E-cadherin expression, indicative of EMT suppression. Given that EMT plays a pivotal role in GBM progression and therapy resistance, the ability of trichodermin to modulate EMT-related proteins suggests that it may have therapeutic potential in limiting GBM progression.

TMZ exerts its cytotoxic effects primarily through DNA damage, particularly via O6-methylguanine lesions, whereas TMZ resistance remains a major limitation in GBM therapy, particularly owing to MGMT-mediated DNA repair mechanisms.^22–24^ Our study demonstrates that trichodermin significantly enhanced TMZ-induced cytotoxicity in GBM cells, as evidenced by the MTT assay. CI analysis confirmed a synergistic interaction between trichodermin and TMZ, suggesting that trichodermin functions as a chemosensitising agent. The combination treatment significantly increased the proportion of apoptotic cells and the sub-G_1_ population of cell cycle, while reducing the proportion of cells in the G_2_/M phase compared with TMZ treatment alone. These findings suggest that trichodermin may enhance TMZ-induced cytotoxicity by promoting a shift from cell cycle arrest to increased apoptotic cell death. Overcoming TMZ resistance remains a critical challenge in GBM treatment, and natural compounds such as trichodermin may provide novel strategies to enhance chemotherapy efficacy. Although the present study provides initial evidence supporting a synergistic interaction at the cellular level, further investigations are required to clarify associated molecular mechanisms, particularly in relation to DNA damage signalling and resistance pathways, such as DNA repair capacity and MGMT-related mechanisms.

Beyond GBM, trichodermin has demonstrated significant anticancer effects in various malignancies through various pathways. In chondrosarcoma, trichodermin induced apoptosis through mitochondrial dysfunction and ER stress, leading to caspase activation.^11^ In pancreatic cancer, trichodermin induced the activation of c-Jun N-terminal kinase (JNK), promoted mitotic arrest and DNA damage, resulting in tumour cell death.^13^ In ovarian cancer, trichodermin inhibited c-Myc expression, leading to G_0_/G_1_ cell cycle arrest and tumour suppression.^14^ A previous study demonstrated that trichodermin suppressed migration and invasion of colorectal cancer cells by moderating the PKC-ERK-Sp1-CTSV pathway.^25^ Additionally, in oral cancer, trichodermin inhibited tumour growth via histone deacetylase-2 (HDAC-2) signalling, further demonstrating its broad-spectrum anticancer properties.^15^ These findings suggest that trichodermin exerts anti-tumour effects by interfering with various pathways, such as mitogen-activated protein kinase (MAPK), across different cancer types. While the present study provides evidence that trichodermin induces apoptosis, modulates cell cycle progression, and suppresses invasion in GBM cells, further investigations are required to delineate the MAPK-mediated mechanisms. To further explore the potential involvement of the PI3K/AKT signalling pathway, we performed preliminary experiments using an AKT inhibitor (MK-2206) and an AKT activator (SC-79). The results of the MTT assay showed that neither inhibition nor activation of AKT significantly altered the trichodermin-induced reduction in cell viability in both T98G and A172 cells (Figure S1), suggesting that this pathway may not play a central role in the observed anticancer effects under the investigated conditions. In addition, given that trichodermin is known to inhibit protein synthesis,^26–28^ it is important to determine whether translational inhibition contributes to the observed anticancer effects in GBM cells. Elucidation of these mechanisms may provide deeper insight into how trichodermin exerts its biological activity.

In the present study, the *in vivo* antitumor activity of trichodermin was evaluated using an orthotopic GBM model. Tumour progression was monitored by bioluminescence imaging, which demonstrated that trichodermin treatment effectively suppressed intracranial tumour growth. At the experimental endpoint, mice treated with the higher dose of trichodermin (0.5 mg/kg/day) exhibited a modest improvement in body weight compared with the control group. In addition, Kaplan–Meier survival analysis revealed a benefit in the trichodermin-treated group compared with the control group, further supporting its therapeutic potential *in vivo*. Histopathological examination by H&E staining demonstrated morphological alterations in tumour tissues following trichodermin treatment, while immunohistochemical staining showed increased cleaved caspase-3 expression in tumours from trichodermin-treated mice. These findings provide *in vivo* evidence of apoptosis induction and are consistent with the caspase-dependent apoptotic effects observed *in vitro*, thereby strengthening the mechanistic link between the cellular and animal studies. Although the present findings support both the efficacy and apoptotic activity of trichodermin *in vivo*, more comprehensive investigations, including detailed toxicological assessment (e.g. organ histology) and pharmacokinetic/pharmacodynamic analyses, are necessary to establish its safety profile and translational applicability.

From a medicinal chemistry perspective, trichodermin belongs to the trichothecene family of sesquiterpenes characterised by a conserved 12,13-epoxytrichothec-9-ene core structure, which is widely recognised as a critical pharmacophore responsible for its biological activity.^29^^,^^30^ Previous studies have demonstrated that structural variations within trichothecene derivatives, including modifications in hydroxylation patterns, epoxide configuration, and side-chain substituents, can influence cytotoxic potency and biological selectivity.^30^ In a previous study, although no chemical derivatization was performed, several structurally related trichothecene analogues were successfully isolated and their cytotoxic activities were compared. Structurally related compounds such as trichodermol, trichoderminol, and trichodermarin derivatives isolated from *Trichoderma brevicompactum* exhibit differential anticancer activities in HCT-116, PC-3, and SK-Hep-1 cell lines.^31^ Compared with these compounds, trichodermin consistently exhibited the strongest anticancer activity, suggesting that its specific structural configuration may play a critical role in its biological potency. Although a detailed structure–activity relationship (SAR) analysis was not performed in the present study, these findings provide preliminary experimental support for structure-dependent activity within the trichothecene scaffold. Future studies focusing on rational modification of functional groups and systematic evaluation of derivative compounds may improve therapeutic selectivity and enhance anticancer efficacy, particularly in the context of glioblastoma.

The broad-spectrum anti-tumour activity of trichodermin highlights its potential as a promising candidate for GBM therapy, either as a monotherapy or in combination with TMZ. By targeting multiple pathways, including cell cycle arrest, apoptosis induction, EMT suppression, and chemosensitisation, trichodermin could complement existing treatments and improve patient outcomes. However, several challenges and limitations of the present study should be acknowledged and addressed before clinical translation. First, the cytotoxicity of trichodermin in normal cells was not evaluated; therefore, the selectivity window remains to be defined. Importantly, a previous study reported that trichodermin exhibits selective cytotoxic effects in ovarian cancer cells compared to normal cells,^14^ supporting its potential as a therapeutic candidate. One of the key considerations is the ability of trichodermin to cross the blood-brain barrier (BBB). A computational prediction using an established BBB prediction model (https://www.ddl.unimi.it/vegaol/bbbp.htm) suggested that trichodermin may possess the ability to penetrate the BBB. Although computational prediction cannot substitute experimental validation, it provides preliminary support for the potential central nervous system accessibility of trichodermin. Given the importance of BBB permeability in glioblastoma therapy, further studies, such as those using *in vitro* BBB models or *in vivo* pharmacokinetic analyses, are necessary to confirm whether trichodermin can effectively penetrate the BBB and exert its therapeutic effects in intracranial tumours. Additionally, structural modifications or nanoparticle-based delivery systems may be necessary to enhance BBB permeability.^32–34^ Future research should also investigate the effects of trichodermin on GBM stem-like cells, which are highly resistant to conventional therapies and contribute to tumour recurrence. Furthermore, comprehensive upstream mechanisms, preclinical toxicology, and pharmacokinetic/pharmacodynamic studies are warranted to assess the long-term safety and potential off-target effects of trichodermin.

## 5. Conclusions

This study demonstrates that trichodermin exerts potent anti-tumour effects against GBM by targeting multiple oncogenic pathways. Trichodermin significantly reduces GBM cell viability, induces cytotoxicity, and inhibits clonogenic potential in a dose- and time-dependent manner. Mechanistically, it disrupts cell cycle progression by inducing G_2_/M phase arrest through the downregulation of cyclin B, cyclin A, and CDK1, thereby preventing tumour cell proliferation. Additionally, trichodermin effectively triggers apoptosis via a caspase-dependent pathway, as evidenced by the cleavage of caspase-3 and PARP, with apoptosis being significantly attenuated upon caspase inhibition. Beyond its cytotoxic effects, trichodermin suppresses GBM cell invasion and cell motility by modulating EMT-associated markers, reducing MMP2 and Snail while promoting E-cadherin expression, suggesting an anti-metastatic role. Moreover, trichodermin enhances the therapeutic efficacy of TMZ, the standard chemotherapy for GBM, by exhibiting a synergistic interaction that potentiates TMZ-induced cell death, particularly at lower drug concentrations. This finding highlights its potential as an adjuvant therapy to overcome chemoresistance. Importantly, *in vivo* studies using a GBM orthotopic mouse model confirm that trichodermin effectively suppresses tumour growth, prolongs survival, and induces caspase 3 cleavage in tumour tissues. Overall, these findings suggest that trichodermin is a bioactive lead compound with anti-glioblastoma activity, with the ability to inhibit tumour progression through cell cycle arrest, apoptosis induction, invasion suppression, and enhancement of TMZ sensitivity. Further mechanistic investigations and preclinical evaluations are warranted to elucidate its clinical potential and optimise its therapeutic applications in glioblastoma treatment.

## Supplementary Material

Supplemental Material

## Data Availability

The datasets generated and analysed during the current study are available from the corresponding author upon reasonable request. All other relevant data supporting the findings of this study are included within the article and its supplementary material.
