# Supplementary material for: Trichodermin exhibits potent anti-glioblastoma activity by inducing cell cycle arrest and apoptosis, suppressing invasion, and enhancing temozolomide efficacy
Source: J Enzyme Inhib Med Chem. 2026 Jul 20;41(1):2694181. doi: 10.1080/14756366.2026.2694181 (PMC13386590; doi:10.1080/14756366.2026.2694181)
Supplement: Supplemental Material [file IENZ_A_2694181_SM0468.zip › IENZ 2694181 - SUPPLEMENTARY FILES/Figure S1.pdf]

Supplemental materials

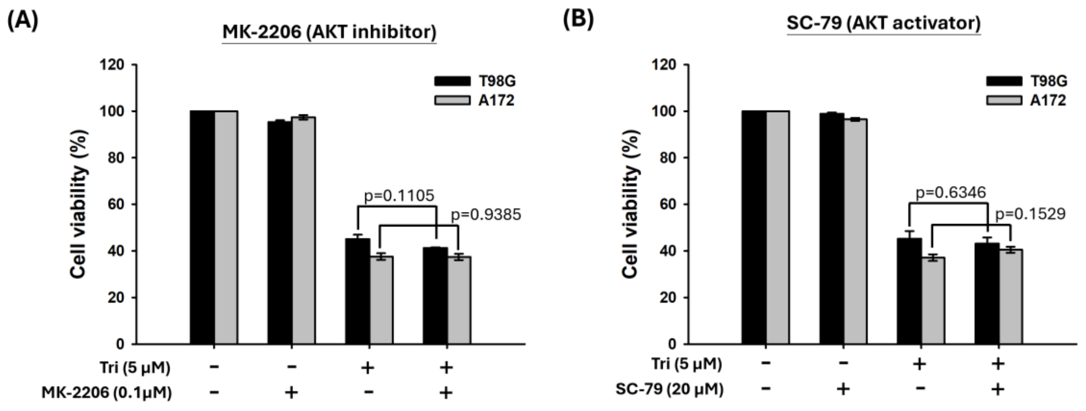

Figure S1. Pharmacological modulation of AKT signalling does not affect trichodermin-induced cytotoxicity in GBM cells. (A and B) T98G and A172 glioblastoma cells were treated with 5 μM trichodermin in the presence or absence of 0.1 μM MK-2206 (AKT inhibitor) (A) or 20 μM SC-79 (AKT activator) (B) for 72 h. Cell viability was assessed using the MTT assay. Data are presented as mean ± SD for n=3. No statistically significant differences are observed between trichodermin treatment alone and co-treatment groups.

Information Classification: General
