# Supplementary material for: Trichodermin exhibits potent anti-glioblastoma activity by inducing cell cycle arrest and apoptosis, suppressing invasion, and enhancing temozolomide efficacy
Source: J Enzyme Inhib Med Chem. 2026 Jul 20;41(1):2694181. doi: 10.1080/14756366.2026.2694181 (PMC13386590; doi:10.1080/14756366.2026.2694181)
Supplement: Supplemental Material [file IENZ_A_2694181_SM0468.zip › IENZ 2694181 - SUPPLEMENTARY FILES/Table S1.pdf]

Figure	assay	cell	treatment	Condition	Replicates	Comparison	p value	Significance
1A	MTT assay	T98G	dose - 24 h	0.5 μM Trichodermin	3	compared with control	0.0013	**
			dose - 24 h	1 μM Trichodermin	3	compared with control	0.0004	***
			dose - 24 h	2.5 μM Trichodermin	3	compared with control	<0.0001	***
			dose - 24 h	5 μM Trichodermin	3	compared with control	<0.0001	***
			dose - 24 h	10 μM Trichodermin	3	compared with control	<0.0001	***
			dose - 24 h	20 μM Trichodermin	3	compared with control	<0.0001	***
			dose - 24 h	40 μM Trichodermin	3	compared with control	<0.0001	***
			dose - 48 h	0.5 μM Trichodermin	3	compared with control	0.0005	***
			dose - 48 h	1 μM Trichodermin	3	compared with control	0.0001	***
			dose - 48 h	2.5 μM Trichodermin	3	compared with control	<0.0001	***
			dose - 48 h	5 μM Trichodermin	3	compared with control	<0.0001	***
			dose - 48 h	10 μM Trichodermin	3	compared with control	<0.0001	***
			dose - 48 h	20 μM Trichodermin	3	compared with control	<0.0001	***
			dose - 48 h	40 μM Trichodermin	3	compared with control	<0.0001	***
			dose - 72 h	0.5 μM Trichodermin	3	compared with control	<0.0001	***
			dose - 72 h	1 μM Trichodermin	3	compared with control	<0.0001	***
			dose - 72 h	2.5 μM Trichodermin	3	compared with control	<0.0001	***
			dose - 72 h	5 μM Trichodermin	3	compared with control	<0.0001	***
			dose - 72 h	10 μM Trichodermin	3	compared with control	<0.0001	***
			dose - 72 h	20 μM Trichodermin	3	compared with control	<0.0001	***

Information Classification: General

			dose - 72 h	40 µM Trichodermin	3	compared with control	<0.0001	***
		A172	dose - 24 h	0.5 µM Trichodermin	3	compared with control	0.0023	**
			dose - 24 h	1 µM Trichodermin	3	compared with control	0.0003	***
			dose - 24 h	2.5 µM Trichodermin	3	compared with control	0.0006	***
			dose - 24 h	5 µM Trichodermin	3	compared with control	0.0007	***
			dose - 24 h	10 µM Trichodermin	3	compared with control	0.0003	***
			dose - 24 h	20 µM Trichodermin	3	compared with control	0.0003	***
			dose - 24 h	40 µM Trichodermin	3	compared with control	<0.0001	***
			dose - 48 h	0.5 µM Trichodermin	3	compared with control	<0.0001	***
			dose - 48 h	1 µM Trichodermin	3	compared with control	<0.0001	***
			dose - 48 h	2.5 µM Trichodermin	3	compared with control	<0.0001	***
			dose - 48 h	5 µM Trichodermin	3	compared with control	0.0001	***
			dose - 48 h	10 µM Trichodermin	3	compared with control	<0.0001	***
			dose - 48 h	20 µM Trichodermin	3	compared with control	<0.0001	***
			dose - 48 h	40 µM Trichodermin	3	compared with control	<0.0001	***
			dose - 72 h	0.5 µM Trichodermin	3	compared with control	<0.0001	***
			dose - 72 h	1 µM Trichodermin	3	compared with control	<0.0001	***
			dose - 72 h	2.5 µM Trichodermin	3	compared with control	0.0003	***
			dose - 72 h	5 µM Trichodermin	3	compared with control	0.0001	***
			dose - 72 h	10 µM Trichodermin	3	compared with control	<0.0001	***
			dose - 72 h	20 µM Trichodermin	3	compared with control	<0.0001	***

Information Classification: General

			dose - 72 h	40 µM Trichodermin	3	compared with control	<0.0001	***
1B	LDH release assay	T98G	dose	0.5 µM Trichodermin	3	compared with control	0.0023	**
			dose	1 µM Trichodermin	3	compared with control	<0.0001	***
			dose	2.5 µM Trichodermin	3	compared with control	<0.0001	***
			dose	5 µM Trichodermin	3	compared with control	0.0001	***
			dose	10 µM Trichodermin	3	compared with control	<0.0001	***
			dose	20 µM Trichodermin	3	compared with control	<0.0001	***
			dose	40 µM Trichodermin	3	compared with control	<0.0001	***
		A172	dose	0.5 µM Trichodermin	3	compared with control	0.6932	ns
			dose	1 µM Trichodermin	3	compared with control	0.0866	ns
			dose	2.5 µM Trichodermin	3	compared with control	<0.0001	***
			dose	5 µM Trichodermin	3	compared with control	0.0001	***
			dose	10 µM Trichodermin	3	compared with control	<0.0001	***
			dose	20 µM Trichodermin	3	compared with control	<0.0001	***
			dose	40 µM Trichodermin	3	compared with control	<0.0001	***
1C	colony formation assay	T98G	dose	0.5 µM Trichodermin	3	compared with control	<0.0001	***
			dose	1 µM Trichodermin	3	compared with control	<0.0001	***
			dose	2.5 µM Trichodermin	3	compared with control	<0.0001	***
			dose	5 µM Trichodermin	3	compared with control	<0.0001	***
		A172	dose	0.5 µM Trichodermin	3	compared with control	<0.0001	***
			dose	1 µM Trichodermin	3	compared with control	<0.0001	***

Information Classification: General

			dose	2.5 µM Trichodermin	3	compared with control	<0.0001	***
			dose	5 µM Trichodermin	3	compared with control	<0.0001	***
2C	Western blot	T98G	dose: p-p53/p53	2.5 µM Trichodermin	3	compared with control	0.0004	***
			dose: p-p53/p53	5 µM Trichodermin	3	compared with control	<0.0001	***
			dose: p-p53/p53	10 µM Trichodermin	3	compared with control	<0.0001	***
			dose: p-p53/p53	20 µM Trichodermin	3	compared with control	<0.0001	***
			dose: p-p53/p53	40 µM Trichodermin	3	compared with control	0.001	**
			dose: Cyclin B	2.5 µM Trichodermin	3	compared with control	<0.0001	***
			dose: Cyclin B	5 µM Trichodermin	3	compared with control	<0.0001	***
			dose: Cyclin B	10 µM Trichodermin	3	compared with control	<0.0001	***
			dose: Cyclin B	20 µM Trichodermin	3	compared with control	<0.0001	***
			dose: Cyclin B	40 µM Trichodermin	3	compared with control	<0.0001	***
			dose: Cyclin A	2.5 µM Trichodermin	3	compared with control	0.0002	***
			dose: Cyclin A	5 µM Trichodermin	3	compared with control	<0.0001	***
			dose: Cyclin A	10 µM Trichodermin	3	compared with control	<0.0001	***
			dose: Cyclin A	20 µM Trichodermin	3	compared with control	<0.0001	***
			dose: Cyclin A	40 µM Trichodermin	3	compared with control	<0.0001	***
			dose: CDK1	2.5 µM Trichodermin	3	compared with control	<0.0001	***
			dose: CDK1	5 µM Trichodermin	3	compared with control	<0.0001	***
			dose: CDK1	10 µM Trichodermin	3	compared with control	<0.0001	***
			dose: CDK1	20 µM Trichodermin	3	compared with control	<0.0001	***

Information Classification: General

			dose: CDK1	40 µM Trichodermin	3	compared with control	<0.0001	***
		A172	dose: p-p53/p53	2.5 µM Trichodermin	3	compared with control	0.0194	*
			dose: p-p53/p53	5 µM Trichodermin	3	compared with control	0.0005	***
			dose: p-p53/p53	10 µM Trichodermin	3	compared with control	0.0006	***
			dose: p-p53/p53	20 µM Trichodermin	3	compared with control	<0.0001	***
			dose: p-p53/p53	40 µM Trichodermin	3	compared with control	<0.0001	***
			dose: Cyclin B	2.5 µM Trichodermin	3	compared with control	0.4361	ns
			dose: Cyclin B	5 µM Trichodermin	3	compared with control	<0.0001	***
			dose: Cyclin B	10 µM Trichodermin	3	compared with control	<0.0001	***
			dose: Cyclin B	20 µM Trichodermin	3	compared with control	<0.0001	***
			dose: Cyclin B	40 µM Trichodermin	3	compared with control	<0.0001	***
			dose: Cyclin A	2.5 µM Trichodermin	3	compared with control	0.0298	*
			dose: Cyclin A	5 µM Trichodermin	3	compared with control	0.0034	**
			dose: Cyclin A	10 µM Trichodermin	3	compared with control	<0.0001	***
			dose: Cyclin A	20 µM Trichodermin	3	compared with control	0.0001	***
			dose: Cyclin A	40 µM Trichodermin	3	compared with control	<0.0001	***
			dose: CDK1	2.5 µM Trichodermin	3	compared with control	<0.0001	***
			dose: CDK1	5 µM Trichodermin	3	compared with control	<0.0001	***
			dose: CDK1	10 µM Trichodermin	3	compared with control	<0.0001	***
			dose: CDK1	20 µM Trichodermin	3	compared with control	<0.0001	***
			dose: CDK1	40 µM Trichodermin	3	compared with control	<0.0001	***

Information Classification: General

2D	Western blot	T98G	time: p-p53/p53	8 h	3	compared with control	<0.0001	***
			time: p-p53/p53	16 h	3	compared with control	0.0004	***
			time: p-p53/p53	24 h	3	compared with control	0.0008	***
			time: p-p53/p53	48 h	3	compared with control	<0.0001	***
			time: p-p53/p53	72 h	3	compared with control	0.0015	**
			time: Cyclin B	8 h	3	compared with control	0.0002	***
			time: Cyclin B	16 h	3	compared with control	<0.0001	***
			time: Cyclin B	24 h	3	compared with control	<0.0001	***
			time: Cyclin B	48 h	3	compared with control	<0.0001	***
			time: Cyclin B	72 h	3	compared with control	<0.0001	***
			time: Cyclin A	8 h	3	compared with control	0.0001	***
			time: Cyclin A	16 h	3	compared with control	<0.0001	***
			time: Cyclin A	24 h	3	compared with control	<0.0001	***
			time: Cyclin A	48 h	3	compared with control	<0.0001	***
			time: Cyclin A	72 h	3	compared with control	<0.0001	***
			time: CDK1	8 h	3	compared with control	0.1267	ns
			time: CDK1	16 h	3	compared with control	0.8943	ns
			time: CDK1	24 h	3	compared with control	0.0016	**
			time: CDK1	48 h	3	compared with control	<0.0001	***
			time: CDK1	72 h	3	compared with control	<0.0001	***
		A172	time: p-p53/p53	8 h	3	compared with control	0.0104	*

Information Classification: General

			time: p-p53/p53	16 h	3	compared with control	0.009	**
			time: p-p53/p53	24 h	3	compared with control	0.0009	***
			time: p-p53/p53	48 h	3	compared with control	0.0013	**
			time: p-p53/p53	72 h	3	compared with control	<0.0001	***
			time: Cyclin B	8 h	3	compared with control	0.0299	*
			time: Cyclin B	16 h	3	compared with control	0.0034	**
			time: Cyclin B	24 h	3	compared with control	<0.0001	***
			time: Cyclin B	48 h	3	compared with control	0.0001	***
			time: Cyclin B	72 h	3	compared with control	<0.0001	***
			time: Cyclin A	8 h	3	compared with control	0.0327	*
			time: Cyclin A	16 h	3	compared with control	0.0195	*
			time: Cyclin A	24 h	3	compared with control	0.0005	***
			time: Cyclin A	48 h	3	compared with control	0.0002	***
			time: Cyclin A	72 h	3	compared with control	0.0004	***
			time: CDK1	8 h	3	compared with control	0.203	ns
			time: CDK1	16 h	3	compared with control	0.0009	***
			time: CDK1	24 h	3	compared with control	<0.0001	***
			time: CDK1	48 h	3	compared with control	0.0003	***
			time: CDK1	72 h	3	compared with control	<0.0001	***
3A	Apoptosis	T98G	dose	2.5 µM Trichodermin	3	compared with control	0.0021	**
			dose	5 µM Trichodermin	3	compared with control	0.0026	**

Information Classification: General

			dose	10 µM Trichodermin	3	compared with control	0.0005	***
			dose	20 µM Trichodermin	3	compared with control	<0.0001	***
			dose	40 µM Trichodermin	3	compared with control	<0.0001	***
		A172	dose	2.5 µM Trichodermin	3	compared with control	0.0003	***
			dose	5 µM Trichodermin	3	compared with control	0.0002	***
			dose	10 µM Trichodermin	3	compared with control	<0.0001	***
			dose	20 µM Trichodermin	3	compared with control	<0.0001	***
			dose	40 µM Trichodermin	3	compared with control	0.0001	***
3B	Apoptosis	T98G	time	24 h	3	compared with control	0.1663	ns
			time	48 h	3	compared with control	0.0017	**
			time	72 h	3	compared with control	<0.0001	***
		A172	time	24 h	3	compared with control	0.1203	ns
			time	48 h	3	compared with control	<0.0001	***
			time	72 h	3	compared with control	<0.0001	***
3F	Apoptosis	T98G	zVAD and Trichodermin combination	zVAD	3	compared with control	0.5927	ns
			zVAD and Trichodermin combination	Trichodermin	3	compared with control	<0.0001	***

Information Classification: General

			zVAD and Trichodermin combination	Combination	3	compared with control	0.0002	***
			zVAD and Trichodermin combination	Combination	3	compared with trichodermin	0.0003	***
		A172	zVAD and Trichodermin combination	zVAD	3	compared with control	0.6741	ns
			zVAD and Trichodermin combination	Trichodermin	3	compared with control	0.0004	***
			zVAD and Trichodermin combination	Combination	3	compared with control	0.1075	ns
			zVAD and Trichodermin combination	Combination	3	compared with trichodermin	0.0027	**
3G	MTT assay	T98G	zVAD and Trichodermin combination	zVAD	3	compared with control	0.4813	ns

Information Classification: General

			zVAD and Trichodermin combination	Trichodermin	3	compared with control	<0.0001	***
			zVAD and Trichodermin combination	Combination	3	compared with control	<0.0001	***
			zVAD and Trichodermin combination	Combination	3	compared with trichodermin	0.0002	***
		A172	zVAD and Trichodermin combination	zVAD	3	compared with control	0.2137	ns
			zVAD and Trichodermin combination	Trichodermin	3	compared with control	<0.0001	***
			zVAD and Trichodermin combination	Combination	3	compared with control	<0.0001	***
			zVAD and Trichodermin combination	Combination	3	compared with trichodermin	0.0395	*
4A	Invation assay	T98G	dose	2.5 μM Trichodermin	3	compared with control	<0.0001	***

Information Classification: General

			dose	5 µM Trichodermin	3	compared with control	<0.0001	***
			dose	10 µM Trichodermin	3	compared with control	<0.0001	***
			dose	20 µM Trichodermin	3	compared with control	<0.0001	***
			dose	40 µM Trichodermin	3	compared with control	<0.0001	***
		A172	dose	2.5 µM Trichodermin	3	compared with control	<0.0001	***
			dose	5 µM Trichodermin	3	compared with control	<0.0001	***
			dose	A172+H210:I228	3	compared with control	<0.0001	***
			dose	20 µM Trichodermin	3	compared with control	<0.0001	***
			dose	40 µM Trichodermin	3	compared with control	<0.0001	***
4B	Wound healing assay	T98G	Control	6 h	3	compared with control	0.0047	**
			Control	12 h	3	compared with control	0.0001	***
			Control	24 h	3	compared with control	<0.0001	***
			Control	30 h	3	compared with control	<0.0001	***
			1 µM	6 h	3	compared with control	0.043	*
			1 µM	12 h	3	compared with control	0.0061	**
			1 µM	24 h	3	compared with control	0.0004	***
			1 µM	30 h	3	compared with control	<0.0001	***
			5 µM	6 h	3	compared with control	0.1881	ns
			5 µM	12 h	3	compared with control	0.0294	*
			5 µM	24 h	3	compared with control	0.0052	**
			5 µM	30 h	3	compared with control	0.0064	**

Information Classification: General

			1 μM	6 h	3	compared with control-6 h	0.3542	ns
			1 μM	12 h	3	compared with control-12 h	0.021	*
			1 μM	24 h	3	compared with control-24 h	0.0023	**
			1 μM	30 h	3	compared with control-30 h	0.0009	***
			5 μM	6 h	3	compared with control-6 h	0.5089	ns
			5 μM	12 h	3	compared with control-12 h	0.0299	*
			5 μM	24 h	3	compared with control-24 h	0.0002	***
			5 μM	30 h	3	compared with control-30 h	<0.0001	***
		A172	Control	6 h	3	compared with control	0.0026	**
			Control	12 h	3	compared with control	0.0007	***
			Control	24 h	3	compared with control	<0.0001	***
			Control	30 h	3	compared with control	<0.0001	***
			1 μM	6 h	3	compared with control	0.0017	**
			1 μM	12 h	3	compared with control	0.0002	***

Information Classification: General

			1 µM	24 h	3	compared with control	<0.0001	***
			1 µM	30 h	3	compared with control	0.0009	***
			5 µM	6 h	3	compared with control	0.0001	***
			5 µM	12 h	3	compared with control	<0.0001	***
			5 µM	24 h	3	compared with control	<0.0001	***
			5 µM	30 h	3	compared with control	0.0002	***
			1 µM	6 h	3	compared with control-6 h	0.6275	ns
			1 µM	12 h	3	compared with control-12 h	0.0853	ns
			1 µM	24 h	3	compared with control-24 h	0.004	**
			1 µM	30 h	3	compared with control-30 h	0.0003	***
			5 µM	6 h	3	compared with control-6 h	0.35	ns
			5 µM	12 h	3	compared with control-12 h	0.0177	*
			5 µM	24 h	3	compared with control-24 h	<0.0001	***
			5 µM	30 h	3	compared with control-30 h	<0.0001	***

Information Classification: General

4C	adhesion assay	T98G	1 h	2.5 µM Trichodermin	3	compared with control	<0.0001	***
			1 h	5 µM Trichodermin	3	compared with control	<0.0001	***
			1 h	10 µM Trichodermin	3	compared with control	<0.0001	***
			1 h	20 µM Trichodermin	3	compared with control	<0.0001	***
			1 h	40 µM Trichodermin	3	compared with control	<0.0001	***
			24 h	2.5 µM Trichodermin	3	compared with control	<0.0001	***
			24 h	5 µM Trichodermin	3	compared with control	<0.0001	***
			24 h	10 µM Trichodermin	3	compared with control	<0.0001	***
			24 h	20 µM Trichodermin	3	compared with control	<0.0001	***
			24 h	40 µM Trichodermin	3	compared with control	<0.0001	***
		A172	1 h	2.5 µM Trichodermin	3	compared with control	<0.0001	***
			1 h	5 µM Trichodermin	3	compared with control	<0.0001	***
			1 h	10 µM Trichodermin	3	compared with control	<0.0001	***
			1 h	20 µM Trichodermin	3	compared with control	<0.0001	***
			1 h	40 µM Trichodermin	3	compared with control	<0.0001	***
			24 h	2.5 µM Trichodermin	3	compared with control	<0.0001	***
			24 h	5 µM Trichodermin	3	compared with control	<0.0001	***
			24 h	10 µM Trichodermin	3	compared with control	<0.0001	***
			24 h	20 µM Trichodermin	3	compared with control	<0.0001	***
			24 h	40 µM Trichodermin	3	compared with control	<0.0001	***
4D	Western blot	T98G	dose: MMP2	2.5 µM Trichodermin	3	compared with control	<0.0001	***

Information Classification: General

			dose: MMP2	5 µM Trichodermin	3	compared with control	<0.0001	***
			dose: MMP2	10 µM Trichodermin	3	compared with control	<0.0001	***
			dose: MMP2	20 µM Trichodermin	3	compared with control	<0.0001	***
			dose: MMP2	40 µM Trichodermin	3	compared with control	<0.0001	***
			dose: Snail	2.5 µM Trichodermin	3	compared with control	0.003	**
			dose: Snail	5 µM Trichodermin	3	compared with control	0.0001	***
			dose: Snail	10 µM Trichodermin	3	compared with control	<0.0001	***
			dose: Snail	20 µM Trichodermin	3	compared with control	<0.0001	***
			dose: Snail	40 µM Trichodermin	3	compared with control	<0.0001	***
			dose: N-cadherin	2.5 µM Trichodermin	3	compared with control	0.009	**
			dose: N-cadherin	5 µM Trichodermin	3	compared with control	<0.0001	***
			dose: N-cadherin	10 µM Trichodermin	3	compared with control	0.0002	***
			dose: N-cadherin	20 µM Trichodermin	3	compared with control	0.0015	**
			dose: N-cadherin	40 µM Trichodermin	3	compared with control	<0.0001	***
			dose: E-cadherin	2.5 µM Trichodermin	3	compared with control	0.0031	**
			dose: E-cadherin	5 µM Trichodermin	3	compared with control	0.0012	**
			dose: E-cadherin	10 µM Trichodermin	3	compared with control	<0.0001	***
			dose: E-cadherin	20 µM Trichodermin	3	compared with control	0.0142	*
			dose: E-cadherin	40 µM Trichodermin	3	compared with control	0.0008	***
		A172	dose: MMP2	2.5 µM Trichodermin	3	compared with control	<0.0001	***
			dose: MMP2	5 µM Trichodermin	3	compared with control	<0.0001	***

Information Classification: General

			dose: MMP2	10 μM Trichodermin	3	compared with control	<0.0001	***
			dose: MMP2	20 μM Trichodermin	3	compared with control	<0.0001	***
			dose: MMP2	40 μM Trichodermin	3	compared with control	<0.0001	***
			dose: Snail	2.5 μM Trichodermin	3	compared with control	<0.0001	***
			dose: Snail	5 μM Trichodermin	3	compared with control	<0.0001	***
			dose: Snail	10 μM Trichodermin	3	compared with control	<0.0001	***
			dose: Snail	20 μM Trichodermin	3	compared with control	<0.0001	***
			dose: Snail	40 μM Trichodermin	3	compared with control	<0.0001	***
			dose: N-cadherin	2.5 μM Trichodermin	3	compared with control	0.0007	***
			dose: N-cadherin	5 μM Trichodermin	3	compared with control	<0.0001	***
			dose: N-cadherin	10 μM Trichodermin	3	compared with control	0.0004	***
			dose: N-cadherin	20 μM Trichodermin	3	compared with control	0.0007	***
			dose: N-cadherin	40 μM Trichodermin	3	compared with control	0.0004	***
			dose: E-cadherin	2.5 μM Trichodermin	3	compared with control	0.0126	*
			dose: E-cadherin	5 μM Trichodermin	3	compared with control	0.0015	**
			dose: E-cadherin	10 μM Trichodermin	3	compared with control	0.0002	***
			dose: E-cadherin	20 μM Trichodermin	3	compared with control	0.0076	**
			dose: E-cadherin	40 μM Trichodermin	3	compared with control	0.0007	***
4E	Western blot	T98G	time: MMP2	8 h	3	compared with control	<0.0001	***
			time: MMP2	16 h	3	compared with control	<0.0001	***
			time: MMP2	24 h	3	compared with control	<0.0001	***

Information Classification: General

			time: MMP2	48 h	3	compared with control	<0.0001	***
			time: MMP2	72 h	3	compared with control	<0.0001	***
			time: Snail	8 h	3	compared with control	<0.0001	***
			time: Snail	16 h	3	compared with control	<0.0001	***
			time: Snail	24 h	3	compared with control	<0.0001	***
			time: Snail	48 h	3	compared with control	<0.0001	***
			time: Snail	72 h	3	compared with control	<0.0001	***
			time: N-cadherin	8 h	3	compared with control	0.0003	***
			time: N-cadherin	16 h	3	compared with control	0.0002	***
			time: N-cadherin	24 h	3	compared with control	0.005	**
			time: N-cadherin	48 h	3	compared with control	<0.0001	***
			time: N-cadherin	72 h	3	compared with control	0.0003	***
			time: E-cadherin	8 h	3	compared with control	<0.0001	***
			time: E-cadherin	16 h	3	compared with control	0.0005	***
			time: E-cadherin	24 h	3	compared with control	0.0008	***
			time: E-cadherin	48 h	3	compared with control	<0.0001	***
			time: E-cadherin	72 h	3	compared with control	0.0015	**
		A172	time: MMP2	8 h	3	compared with control	0.0251	*
			time: MMP2	16 h	3	compared with control	0.0099	**
			time: MMP2	24 h	3	compared with control	<0.0001	***
			time: MMP2	48 h	3	compared with control	0.0002	***

Information Classification: General

			time: MMP2	72 h	3	compared with control	<0.0001	***
			time: Snail	8 h	3	compared with control	<0.0001	***
			time: Snail	16 h	3	compared with control	<0.0001	***
			time: Snail	24 h	3	compared with control	<0.0001	***
			time: Snail	48 h	3	compared with control	<0.0001	***
			time: Snail	72 h	3	compared with control	<0.0001	***
			time: N-cadherin	8 h	3	compared with control	0.0132	*
			time: N-cadherin	16 h	3	compared with control	<0.0001	***
			time: N-cadherin	24 h	3	compared with control	<0.0001	***
			time: N-cadherin	48 h	3	compared with control	<0.0001	***
			time: N-cadherin	72 h	3	compared with control	<0.0001	***
			time: E-cadherin	8 h	3	compared with control	0.0104	*
			time: E-cadherin	16 h	3	compared with control	0.009	**
			time: E-cadherin	24 h	3	compared with control	0.001	**
			time: E-cadherin	48 h	3	compared with control	0.0013	**
			time: E-cadherin	72 h	3	compared with control	<0.0001	***
5A	MTT assay	T98G	TMZ and trichodermin	100 μM TMZ	3	compared with control	0.0039	**
			TMZ and trichodermin	200 μM TMZ	3	compared with control	<0.0001	***
			TMZ and trichodermin	400 μM TMZ	3	compared with control	<0.0001	***

Information Classification: General

			TMZ and trichodermin	800 µM TMZ	3	compared with control	<0.0001	***
			TMZ and trichodermin	1000 µM TMZ	3	compared with control	<0.0001	***
			TMZ+1 µM trichodermin	100 µM TMZ	3	compared with control	<0.0001	***
			TMZ+1 µM trichodermin	200 µM TMZ	3	compared with control	<0.0001	***
			TMZ+1 µM trichodermin	400 µM TMZ	3	compared with control	<0.0001	***
			TMZ+1 µM trichodermin	800 µM TMZ	3	compared with control	<0.0001	***
			TMZ+1 µM trichodermin	1000 µM TMZ	3	compared with control	<0.0001	***
			TMZ+2.5 µM trichodermin	100 µM TMZ	3	compared with control	<0.0001	***
			TMZ+2.5 µM trichodermin	200 µM TMZ	3	compared with control	<0.0001	***
			TMZ+2.5 µM trichodermin	400 µM TMZ	3	compared with control	<0.0001	***
			TMZ+2.5 µM trichodermin	800 µM TMZ	3	compared with control	<0.0001	***

Information Classification: General

			TMZ+2.5 µM trichodermin	1000 µM TMZ	3	compared with control	<0.0001	***
			TMZ+5 µM trichodermin	100 µM TMZ	3	compared with control	<0.0001	***
			TMZ+5 µM trichodermin	200 µM TMZ	3	compared with control	<0.0001	***
			TMZ+5 µM trichodermin	400 µM TMZ	3	compared with control	<0.0001	***
			TMZ+5 µM trichodermin	800 µM TMZ	3	compared with control	<0.0001	***
			TMZ+5 µM trichodermin	1000 µM TMZ	3	compared with control	<0.0001	***
5B	MTT assay	A172	TMZ	100 µM TMZ	3	compared with control	0.0311	*
			TMZ	200 µM TMZ	3	compared with control	<0.0001	***
			TMZ	400 µM TMZ	3	compared with control	<0.0001	***
			TMZ	800 µM TMZ	3	compared with control	<0.0001	***
			TMZ	1000 µM TMZ	3	compared with control	<0.0001	***
			TMZ+1 µM trichodermin	100 µM TMZ	3	compared with control	<0.0001	***
			TMZ+1 µM trichodermin	200 µM TMZ	3	compared with control	<0.0001	***

Information Classification: General

			TMZ+1 μM trichodermin	400 μM TMZ	3	compared with control	<0.0001	***
			TMZ+1 μM trichodermin	800 μM TMZ	3	compared with control	<0.0001	***
			TMZ+1 μM trichodermin	1000 μM TMZ	3	compared with control	<0.0001	***
			TMZ+2.5 μM trichodermin	100 μM TMZ	3	compared with control	<0.0001	***
			TMZ+2.5 μM trichodermin	200 μM TMZ	3	compared with control	<0.0001	***
			TMZ+2.5 μM trichodermin	400 μM TMZ	3	compared with control	<0.0001	***
			TMZ+2.5 μM trichodermin	800 μM TMZ	3	compared with control	<0.0001	***
			TMZ+2.5 μM trichodermin	1000 μM TMZ	3	compared with control	<0.0001	***
			TMZ+5 μM trichodermin	100 μM TMZ	3	compared with control	<0.0001	***
			TMZ+5 μM trichodermin	200 μM TMZ	3	compared with control	<0.0001	***
			TMZ+5 μM trichodermin	400 μM TMZ	3	compared with control	<0.0001	***

Information Classification: General

			TMZ+5 μM trichodermin	800 μM TMZ	3	compared with control	<0.0001	***
			TMZ+5 μM trichodermin	1000 μM TMZ	3	compared with control	<0.0001	***
5E	Apoptosis	T98G	TMZ+trichodermin	TMZ+trichodermin	3	compared with control	0.0002	***
			TMZ+trichodermin	TMZ+trichodermin	3	compared with control	0.001	**
			TMZ+trichodermin	TMZ+trichodermin	3	compared with control	<0.0001	***
			TMZ+trichodermin	TMZ+trichodermin	3	compared with TMZ	<0.0001	***
		A172	TMZ+trichodermin	TMZ+trichodermin	3	compared with control	<0.0001	***
			TMZ+trichodermin	TMZ+trichodermin	3	compared with control	<0.0001	***
			TMZ+trichodermin	TMZ+trichodermin	3	compared with control	<0.0001	***
			TMZ+trichodermin	TMZ+trichodermin	3	compared with TMZ	<0.0001	***

Table S1. Exact p-values for statistical comparisons

Notes: p-values were generated from analyses described in the manuscript. Significance codes: * $p < 0.05$; ** $p < 0.01$; *** $p < 0.001$; ns, not significant. Comparisons are listed in the Comparison column.

Information Classification: General
